# Versatile twin layer macromolecular fibers for advanced tissue engineering applications

**DOI:** 10.1016/j.isci.2026.116480

**Published:** 2026-09-10

**Authors:** Caroline S. Taylor, Hamta Majd, David A. Gregory, Annabelle Fricker, Syed Mohammad Daniel Syed Mohamed, Jonathan Hinchcliffe, Emmanuel Asare, Nicholas T. Farr, Hussain Alenezi, Gavin I. Welsh, Mohan Edirisinghe, Ipsita Roy

**Affiliations:** 1 School of Chemical, Materials and Biological Engineering, The University of Sheffield, Sheffield S3 7HQ, UK; 2 Department of Mechanical Engineering, University College London, London WC1E 7JE, UK; 3 Insigneo Institute, The Pam Liversidge Building, University of Sheffield, Sheffield S1 3JD, UK; 4 Bristol Renal, Translational Health Sciences, Bristol Medical School, University of Bristol, Bristol BS1 3NY, UK; 5 Department of Manufacturing Engineering, College of Technological Studies, PAAET, Kuwait City 13092, Kuwait; 6 PHAsT Ltd., The Innovation Centre, 217 Portobello, Sheffield S1 4DP, UK

**Keywords:** PHA, PLA, twin layer, core-sheath, fiber, tissue engineering, pressurized gyration

## Abstract

Finding the ideal biomaterial with all required properties to replace specific tissues is an unmet challenge. In this study, twin-layered fibers with two well-established medical biomaterials, PHAs and PLA, have been produced via pressurized gyration and characterized thoroughly for surface properties, mechanical properties, and *in vitro* cell responses using a range of cell types for both hard and soft tissue engineering. The diameters of fibers produced ranged from 0.9 to 1.2 μm. The PHA sheath fibers increased cell attachment of live viable cells compared to PLA sheath fibers, due to their relatively higher biocompatibility, increased surface roughness, and protein adsorption. PLA, as a core, increased the stiffness and tensile strength of the fibers. This approach produced fibers in a cost-effective, scalable, and sustainable manner, providing a highly effective solution to the challenging clinical need of the regeneration of tissues.

## Highlights

• PHA as a sheath material of fiber improved biocompatibility of fibers
• PLA as core material of fiber significantly increased mechanical properties
• C-S PLA: PHA fibers increased neurite outgrowth lengths of NG108-15 neuronal cells
• C-S PLA: PHA fibers increased mineralization deposits from human primary osteoblasts

## Graphical abstract

**Figure:**
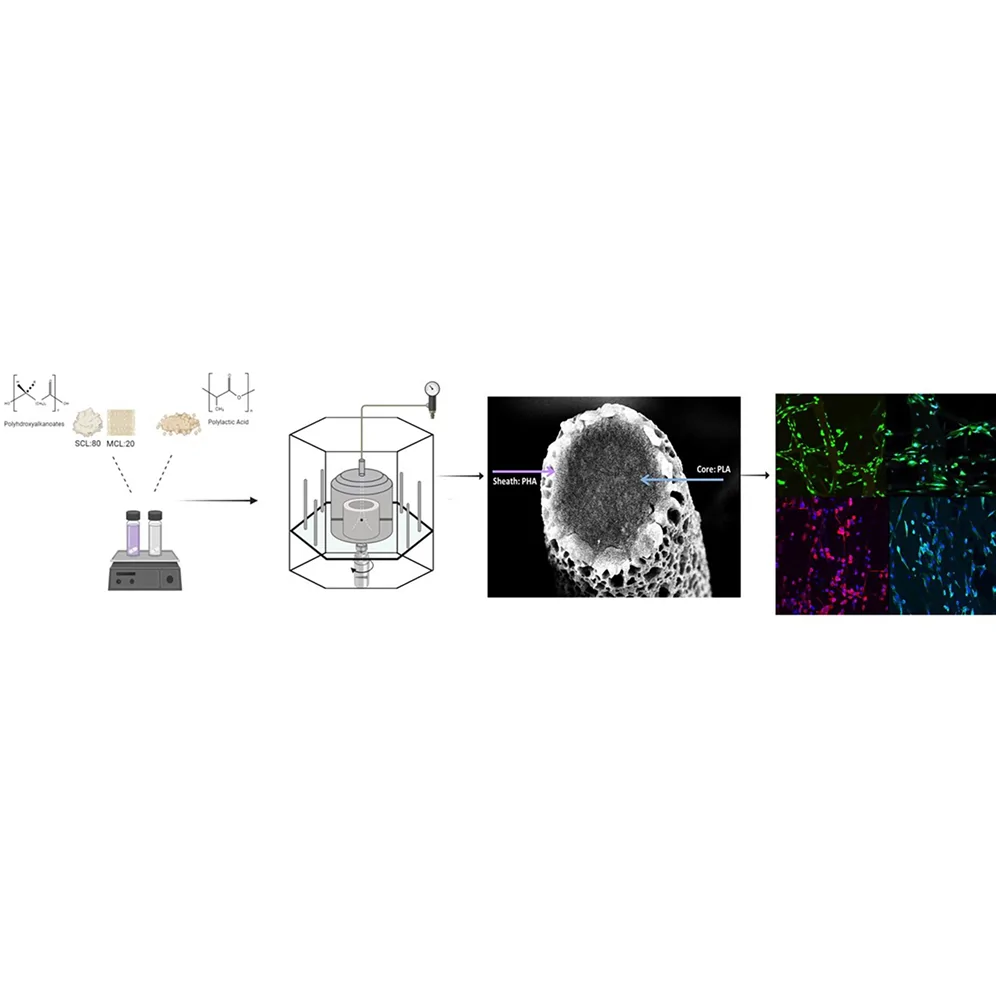

## Teaser

Bioengineering; Material synthesis

## Introduction

Tissue engineering is at the forefront of replacing and regenerating diseased and damaged tissues and organs, using the patient’s own cells and growing them on 3D scaffolds, produced using biomaterials, mimicking the extracellular matrix (ECM) of the tissue.^1^ Due to a lack of organs for transplantation, there is an unmet clinical need for the replacement of tissues and organs, using tissue engineering, and therefore, an increased demand of 3D scaffolds, produced using biomaterials.^2^ Polymer fibers, produced from natural and synthetic biomaterials, offer the most potential as tissue engineering scaffolds, mimicking the fibrous architecture of the ECM, as well as having a porous microstructure and a high surface area to volume ratio that favors cell adhesion, proliferation and differentiation.^3^ However, fiber scaffolds produced from one material, often do not replicate the complexity of different biological systems, such as differences in biocompatibility and mechanical properties, depending on the chosen biomaterial.^4^ Therefore, the combination of using two different polymers, in the form of twin layer fibers, or core-sheath (C-S) fibers, has emerged as an innovative approach to addressing these issues.^5^ By using two polymer materials in a twin-layered structure with a polymer in the core of the fiber and an outer layer, a sheath, i.e., a C-S structure, the physical/mechanical properties of the twin-layered fiber can be tuned with the core material providing the mechanical requirements, and bioactivity/functionality provided by the sheath material, thereby producing tailorable fibers for both hard and soft tissues.^6^ C-S fibers can reflect biomimetic principles by emulating natural hierarchical structures and cell-guiding microstructures, mimicking the ECM of tissues.^7^ Various innovative methods, including coaxial electrospinning and coaxial centrifugal spinning, are being developed in the market for large-scale C-S fiber production and increased manufacturing efficiency, due to high demand from the medical industry.^5^ However, they all have major limitations, such as fiber morphology control, slow production rate, and low production yield.^4^ A recent development to overcome current fiber production challenges is the C-S pressurized gyration (C-S PG), a low-cost technology that can mass-produce smart fibers, for healthcare applications in a more sustainable and scalable way, compared to electrospinning, which requires high-voltage electric fields and is sensitive to humidity.^4^ C-S PG operates by the combination of pressure and centrifugal force to mass-produce C-S fibers in the range of nano to micro scale using a variety of different biomaterials.^8^ Furthermore, C-S PG has many advantages over conventional techniques such as wet spinning, microfluidic spinning, and 3D printing. PG can produce fibers from submicron to several microns, much finer than those produced from wet spinning or 3D printing.^9^^,^^10^ It allows tuning of fiber alignment, porosity, and surface texture, important factors for cell behavior, by adjusting pressure, rotational speed, and polymer concentration. Porosity of scaffolds is more controllable using PG, compared to wet spinning, microfluidics, electrospinning, and 3D printing.^4^ It operates at a continuous, reproducible, and scalable setup. Compared to its counterparts, it avoids the need for a coagulation bath (wet spinning),^11^ microchannel fabrication (microfluidics)^12^ or layer-by-layer programming (3D printing),^13^ reducing variability and complexity of the setup. CS-PG has the potential to solve a huge unmet need in tissue and organ regeneration.

This study investigates the production and assessment of C-S fibers, using C-S PG, with a specific focus on the combination of polyhydroxyalkanoates (PHAs) and polylactic acid (PLA) in a C-S structure, for both hard and soft tissue engineering applications. PLA is a bioresorbable, biocompatible biopolymer, produced from renewable sources, such as corn starch and sugar cane.^14^ It exhibits high stiffness and tensile strength, making it a suitable polymer for applications in bone tissue engineering.^15^ However, PLA degrades quickly resulting in unstable structures, due to bulk erosion, into acidic degradation products, such as lactic acid, limiting its maximal performance in tissue engineering applications.^7^^,^^16^^,^^17^ PHAs, however, are a family of naturally derived polyesters with a selectable range of biocompatible and mechanical properties, making them very attractive materials for tissue engineering.^18^ They are biodegradable, degrading via controllable surface erosion into less acidic degradation products that are natural metabolites, and are sustainably produced by a wide range of microorganisms, eliminating environmental concerns associated with other biopolymers.^19^^,^^20^ PHAs have been extensively investigated in tissue engineering applications in bone, cardiac, nerve, and skin tissue repair,^21^^,^^22^^,^^23^^,^^24^ as well as drug delivery systems. For example, Poly(3-hydroxybutyrate-co-3-hydroxyvalerate-co-3-hydroxyhexanoate) (PHBVHHx) nanoparticles encapsulating ritlecitinib were used to promote angiogenesis in hair follicles, to promote new hair growth.^17^ Additionally, poly(3-hydroxybutyrate-co-3-hydroxyvalerate-co-3-hydroxyhexanoate) (PBVHx) nanoparticles with soybean lecithin-modified BMP4 (sBP-NPs) have been shown to promote osteogenic differentiation of human bone marrow mesenchymal stem cells for bone regeneration applications.^25^

Our previous work investigated PHA blend fibers, produced from PG, for various tissue engineering applications.^26^ Poly(3-hydroxybutyrate) (P(3HB)), a short-chain length (SCL) PHA exhibiting excellent biocompatibility and high mechanical strength, was formed into a blend with Poly(3-hydroxyoctanoate-co-3-hydroxydecanoate) (P(3HO-*co*-3HD)), a medium-chain length (MCL) PHA exhibiting high elastomeric properties, and the resulting fibers proved to be highly effective in cardiac, bone and nerve tissue regeneration.^19^^,^^22^^,^^27^ Therefore, in this study, the PHA blend has been investigated further, in the form of twin-layered C-S fibers, with PLA (Table 1).

**Table 1:** Comparison of single-layer vs. Core-Sheath (PHA/PLA) fiber systems

Property	Single-layer fibers	Core-sheath fibers (PHA/PLA)
Mechanical stability	limited tunability; often brittle or weak	enhanced by combining tough core (e.g., PHA) with ductile sheath (e.g., PLA)^4^
Degradation kinetics	uncontrolled; bulk erosion (e.g., PLA and PCL) or rapid loss via surface erosion (e.g., PCL, enzymatic degradation)	tuneable via sheath/core composition; PHA degrades via surface erosion, enabling predictable profiles^18^
Bioactivity	often bioinert or acidic degradation products. Often requires functionalization to encourage cell attachment.	PHA is bioactive and less inflammatory; degradation products are natural metabolites. Both and core-sheath can be functionalized for specific cell interactions/drug/biologic release and so forth.^18^
Drug/factor delivery	burst release common; limited control	core-sheath allows spatial separation for sustained or staged release^4^
Processability	simpler fabrication, but limited property control	coaxial electrospinning enables tailored architectures and multifunctionality^4^
Application versatility	restricted to narrow use-cases	broader biomedical potential: scaffolds, wound healing, drug delivery^18^

Both PHAs and PLA are considered biomimetic natural biomaterials, mimicking the ECM with biochemical and biophysical cues, tailorable mechanical, bioactivity, and microstructural interconnectivity.^7^ The use of these materials in C-S fibers is hugely advantageous, mimicking the layered architecture of the ECM and mimicking nature’s compartmentalization using different biomimetic materials.^20^

C-S fibers, using PHA and PLA, can significantly overcome the limitations of single-layer systems. We hypothesize that in a C-S configuration, PLA can serve as a reinforcing sheath while PHA provides ductility and resilience in the core.^15^ Degradation rate of the fibers can be controlled by placing PLA as the core material, for quick degradation, or as the sheath material, for slower degradation.^28^ PHAs are inherently more biocompatible and less immunogenic than PLA.^7^ This makes it advantageous as a sheath material, delivering bioactivity directly, or as a core material, interfacing with the tissue, which would not be feasible in single layer systems.^20^ Furthermore, C-S fibers allow for spatial compartmentalization, ideal for dual drug delivery^29^ and gradient scaffolds,^5^ which is not feasible in single fiber systems. The C-S fibers enable the use of the PHA blend, either as the core to decrease mechanical stiffness and strength, or as the sheath component to improve biocompatibility and enhance cell adhesion, with tailored degradation rates, allowing the achievement of a degradation rate ideal for the natural healing process.^26^ The combination of these two polymers in such structures has significant potential in forming scaffolds with tailored physical and biological properties, thus simulating the complexity of the natural ECM.

C-S fibers, produced from the PHA blend and PLA, were investigated for *in vitro* responses using a large range of cell types for hard and soft tissue engineering applications, including nerve, cardiac, bone, pancreas, kidney, and skin tissue cells.^18^ This study thus expands its application to include cell types that have never been explored with such polymer combinations, such as kidney and pancreas cells.^30^^,^^31^ Therefore, a range of scaffolds, can be manufactured, that can efficiently integrate into multiple tissue environments, opening the path for a wide range of regenerative applications, such as bone implants, cardiac patches, stents and blood vessels, sutures, wound dressings, nerve conduits, artificial pancreas, artificial glomerulus, cartilage implants and drug delivery systems.^18^ This study investigates the C-S PG technology to mass-produce twin-layered C-S fibers, consisting of PLA and PHAs as both core and sheath materials, for a range of tissue engineering applications, offering low-cost biomaterial scaffold manufacture, tailored for its application, produced in a cost effective, scalable and sustainable way.

## Results

### Confirmation of twin-layered core-sheath fiber structure

**Figure 1:**
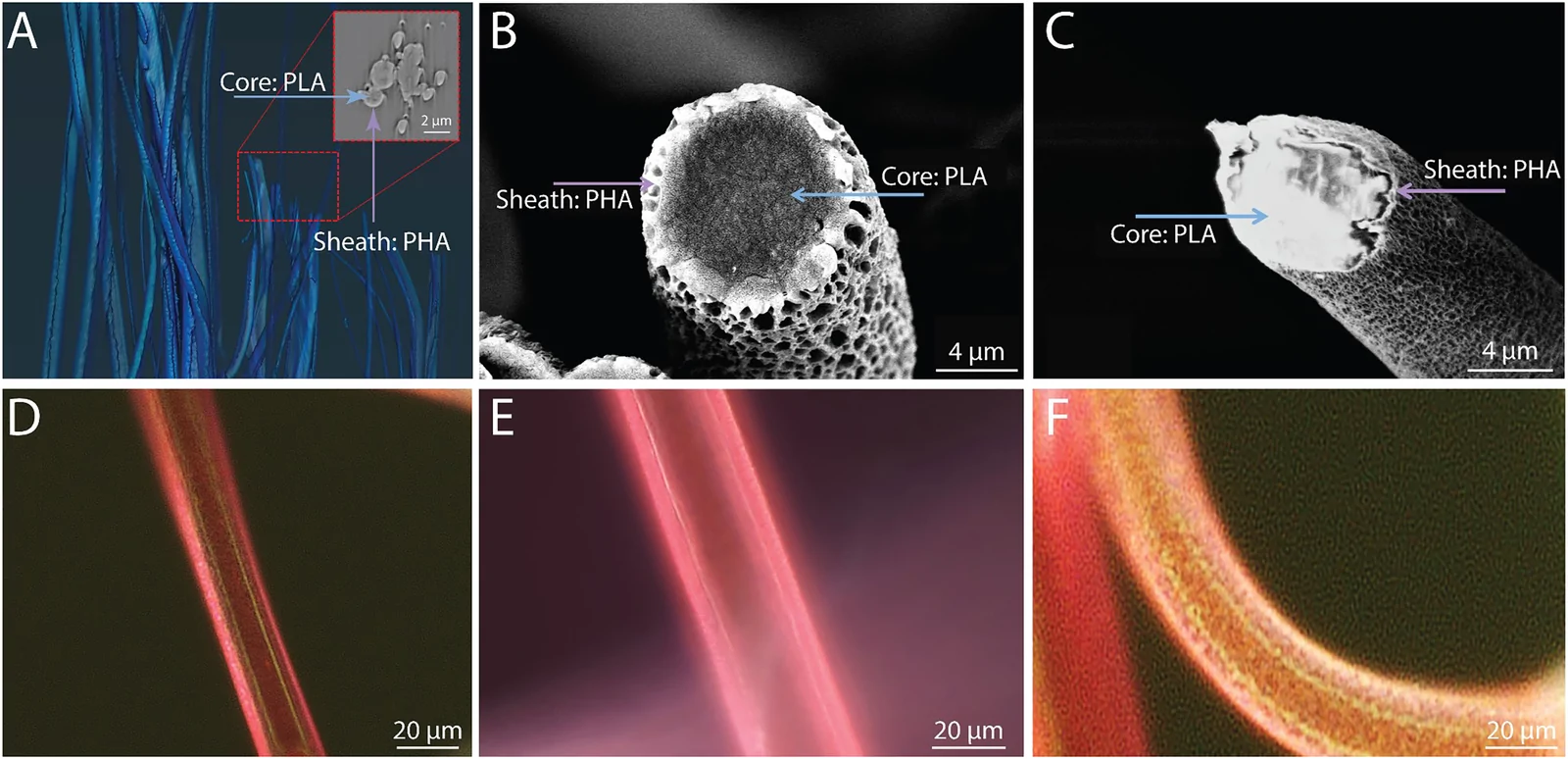
Core–sheath architecture of PLA:PHA fibres (A) In-line phase contrast tomography micrograph of C-S PLA:PHA fibres showing different regions of PLA and PHA in the fibre. (B) SEM Cross-section images of C-S PLA:PHA and (C) C-S PLA:PHA fibres, showing the different regions of PLA and PHA in the fibre. (D–F) Optical migraphs of C-S PLA:PHA fibres at different magnifications. Dark inner region indicates the PLA core and the brighter outer region indicates the PHA sheath.

Figure 1A demonstrates the cross-sectional view of C-S PLA:PHA fibers using in-line phase contrast tomography, in which the sheath layer can be visualized, with a thicker fiber core, as quantified in Figure S3. Figures 1B and 1C demonstrate the presence of a separate sheath and separate core of the C-S PLA:PHA fibers visualized by SEM and Figures 1D and 1E using optical microscopy and fluorescence dyes.

### Characterization of core-sheath fibers

**Figure 2:**
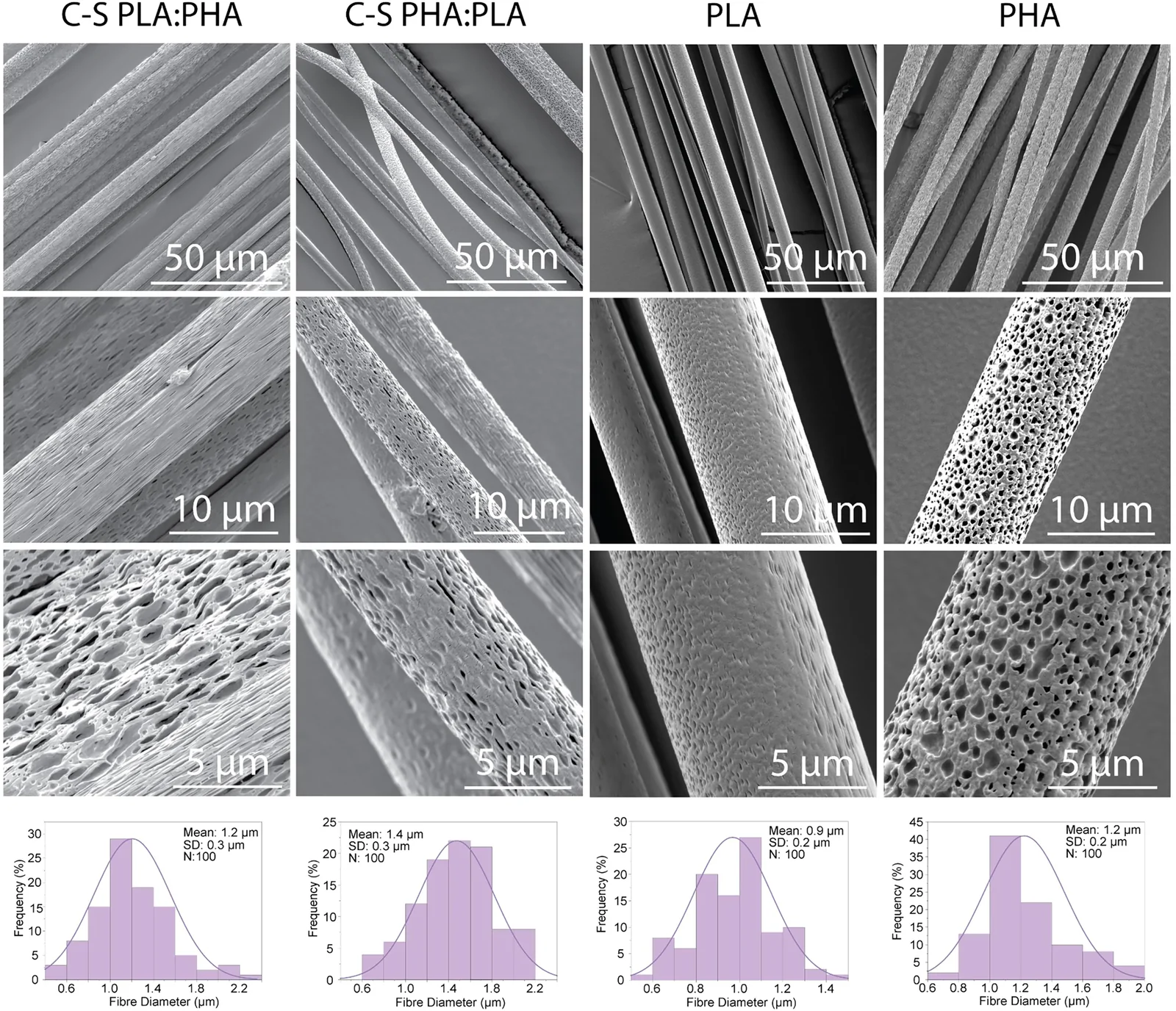
SEM images and size distribution graphs of twin-layered C-S PLA: PHA fibers, C-S PHA:PLA fibers, PLA fibers, and PHA blend fibers (scale bars, 50, 10 and 5 μm, mean ± SD, *n* = 3 independent experiments)

Diameters of C-S fibers produced were determined from SEM images. Fiber diameters of the fibers, fabricated at a working pressure of 0.1 MPa and a constant rotational speed of 6,000 rpm, were 1.2μm ± 0.3 μm and 1.4 μm ± 0.3 μm for the twin-layered C-S PLA:PHA and C-S PHA:PLA, respectively (Figure 2). Mono PLA and PHA blend fibers had diameters of 0.9 μm ± 0.2 μm and 1.2 μm ± 0.2 μm respectively.

### Surface roughness of core-sheath fibers

**Figure 3:**
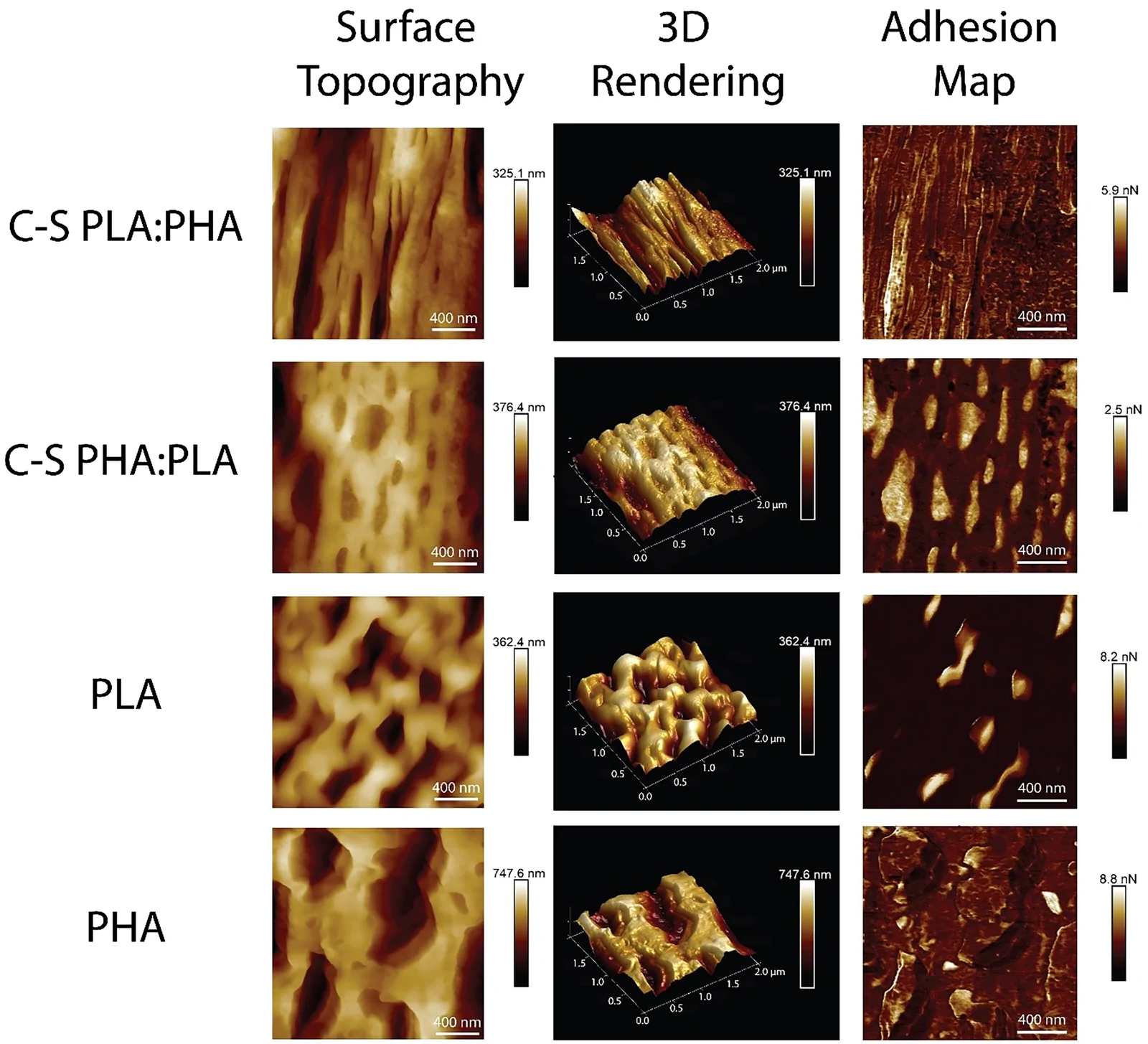
Representative AFM surface topography micrographs, AFM adhesion maps, 3D renderings, and representative histograms show the adhesion distribution of C-S PLA:PHA, C-S PHA:PLA, PLA, and PHA fibers (scale bars, 400 nm, mean ± SD, *n* = 3 independent experiments)

AFM surface topography and 3D rendered micrographs of the twin-layered C-S PLA:PHA, C-S PHA:PLA, PLA, and PHA fibers revealed differences in topography, surface roughness, adhesion, and mechanical properties (Figure 3). The typical root-mean-square (*Rq*) and average roughness (*Ra*) values were calculated from the micrographs obtained, and values are presented in Table 2. As mono-fibers, PHA blend fibers exhibited the highest roughness values, *Rq* value of 187.1 ± 5.2 nm and *Ra* value of 156.5 ± 4.3 nm, compared to the PLA fibers, *Rq* value of 168.5 ± 8.3 nm and *Ra* value of 137.8 ± 1.8 nm. This was also observed with the C-S fibers, in which C-S PLA:PHA fibers exhibited the highest roughness values, indicated by the *Rq* value of 226.3 ± 32.6 nm and *Ra* value of 185.0 ± 26.7 nm, whereas *Rq* and *Ra* values for C-S PHA:PLA fibers were 223.5 ± 39.4 nm and 183.3 ± 34.5 nm, respectively. AFM adhesion maps of the fibers revealed differences in adhesion values, the force measured between the tip of the AFM cantilever and the fiber sample (presented in Table 2). The twin-layered C-S PLA:PHA fibers were more adhesive than the twin-layered C-S PHA:PLA fibers, but not significantly, 2.9 ± 0.2 nN compared to 2.8 ± 2.1 nN, respectively. When observing adhesion on the materials individually, PLA fibers were less adhesive, 1.5 ± 0.4 nN, compared to the PHA fibers, 2.9 ± 0.2 nN. Mono-PHA blend fibers, and PHA blend sheath fibers, exhibited increased porosity, increased surface roughness, and increased protein adsorption (Figure S6), compared to the other fiber conditions.

**Table 2:** *Rq, Ra,* adhesion values, and elastic modulus of core-sheath and mono fibers quantified from images in Figure 3 (mean ± SD, *n* = 3; three replicates per *n* = 1 and three values taken per sample)

Sample	*Rq* (nm)	*Ra* (nm)	Adhesion mean (nN)	DMT modulus (MPa)
C-S PLA:PHA	226.3 ± 32.6	185.0 ± 26.7	2.9 ± 0.2	213. 3 ± 4.3
C-S PHA:PLA	223.5 ± 39.4	183.3 ± 34.5	2.8 ± 2.1	97. 6 ± 3.6
PLA	168.5 ± 8.3	137.8 ± 1.8	1.5 ± 0.4	178.4 ± 5.7
PHA	187. 1 ± 5.2	156. 5 ± 4.3	2.9 ± 0.2	78. 0 ± 2.3

AFM micrographs were used to quantify the surface elastic modulus of the fibers. Young’s modulus was measured using AFM by fitting data into the Derjaguin-Muller-Toporov (DMT) model. C-S PLA:PHA fibers exhibited a significantly higher modulus of 213.3 ± 4.3 MPa, compared to the C-S PHA:PLA fibers which had a modulus of 97. 6 ± 3.6 MPa. Both mono PLA and PHA blend fibers demonstrated Young’s modulus values of 178.4 ± 5.7 and 78. 0 ± 2.3 MPa, respectively.

### NG108-15 neuronal cell differentiation and viability

**Figure 4:**
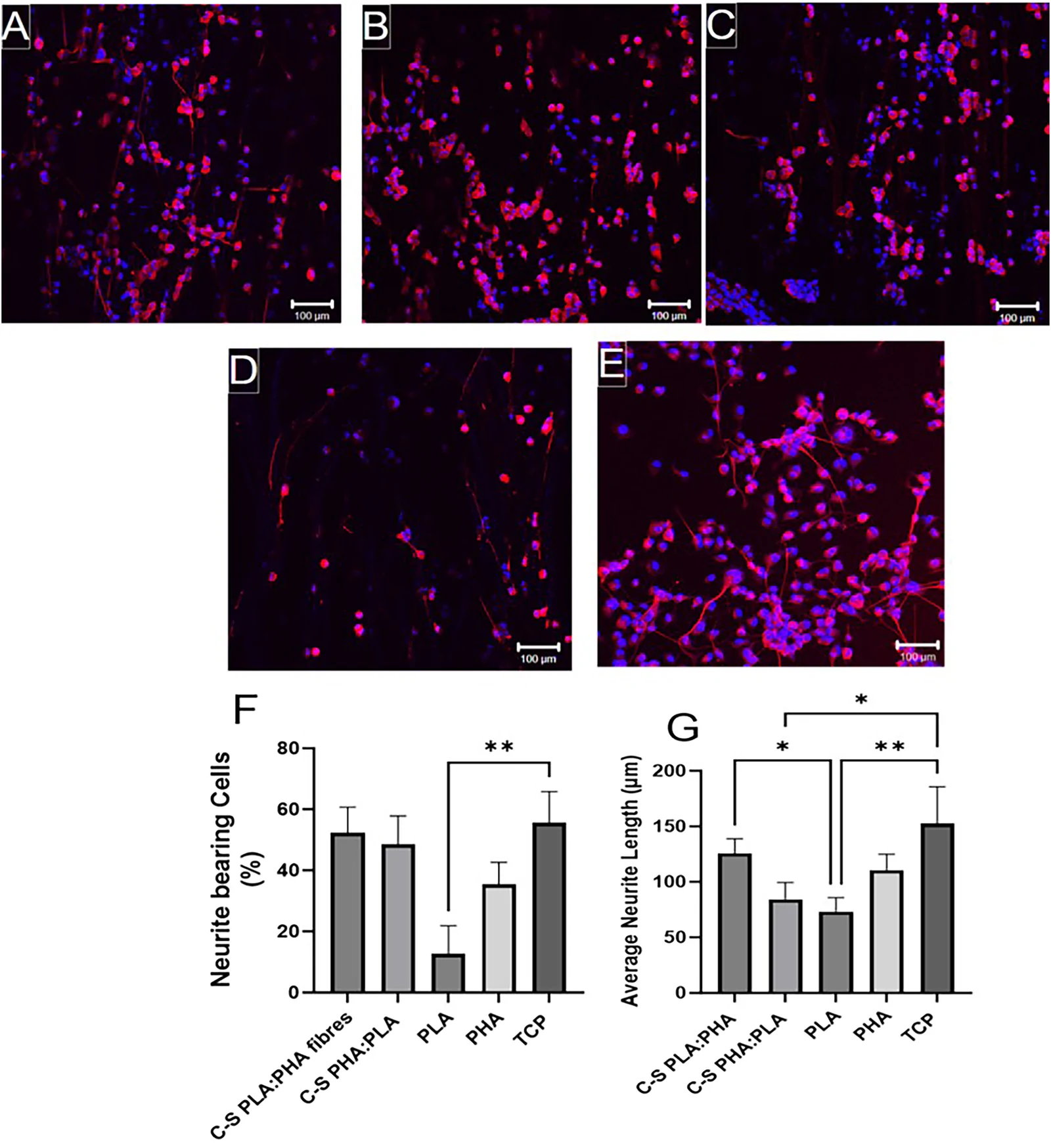
Confocal micrographs of NG108-15 neuronal cells immunolabelled for βIII-tubulin and DAPI on (A) C-S PLA: PHA, (B) C-S PHA: PLA, (C) PLA, and (D) PHA blend fibers. (E) Tissue culture plastic control (scale bars, 100 μm): (F) The percentage of neurite bearing neuronal cells after 6 days in culture; (G) average neurite length per condition after 7 days in culture (mean ± SD, *n* = 3 independent experiments; ∗*p* < 0.05 and ∗∗*p* < 0.01).

NG108-15 neuronal cells stained positively for βIII-tubulin (Figures 4A–4E) and the twin-layered C-S fibers supported NG108-15 neuronal cell differentiation. 52.4 ± 8.3% and 48.5 ± 9.3% of cells exhibited neurite extensions on the twin-layered C-S PLA: PHA and C-S PHA: PLA fibers, respectively (Figure 4F), though no significant differences were detected between the fiber samples. PLA fibers demonstrated a significantly lower number of cells exhibiting neurite extension, 12.6 ± 9.2%, compared to the tissue culture plastic control, 55.62 ± 10.2%. The twin-layered C-S PLA: PHA fibers supported significantly longer NG108-15 neuronal cell neurites, 125.6 ± 13.3 μm, compared to neurites measured on the PLA fibers, 73.1 ± 12.7 μm. The twin-layered C-S PHA: PLA fibers, and PLA fibers demonstrated significantly smaller neurite lengths, of 84.2 ± 15.2 μm and 73.1 ± 12.7 μm, compared to neurites measured on the TCP control (152.4 ± 33.3 μm). No significant difference was detected between the PHA blend fibers, 110.6 ± 14.2 μm, and the twin-layered C-S PLA: PHA fibers, 125.7 ± 13.3 μm.

### C2C12 myoblast cell differentiation

**Figure 5:**
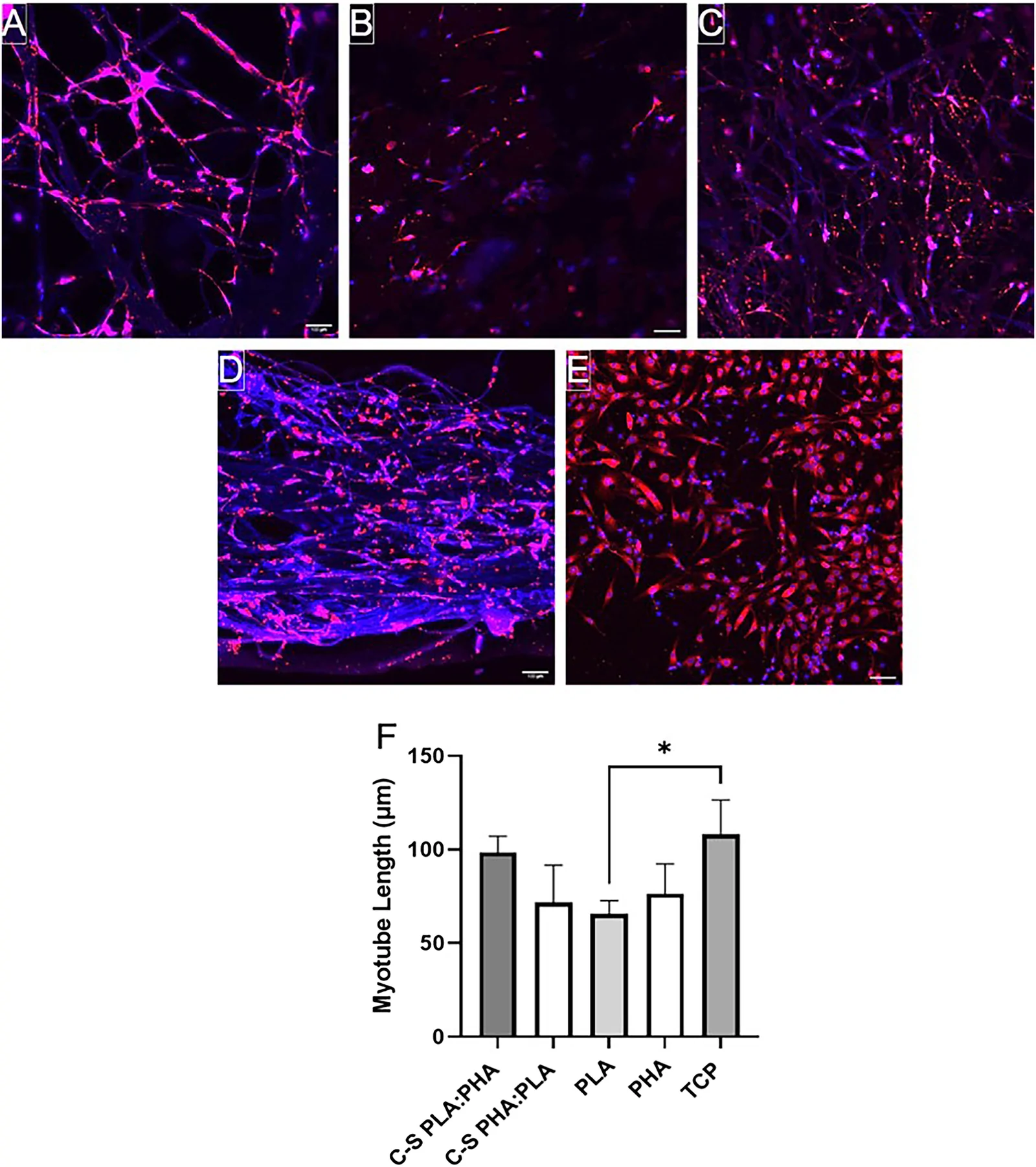
Confocal micrographs of C2C12 myoblast cells stained for myosin (red) and DAPI (blue) on (A) C-S PLA:PHA, (B) C-S PHA:PLA, (C) PLA, and (D) PHA blend fibers. (E) Tissue culture plastic control (scale bars, 100 μm): (F) average myotube length per condition after 7 days in culture (mean ± SD, *n* = 3 independent experiments; ∗*p* < 0.05).

C2C12 myoblast cells stained positively for myosin on all fiber samples (Figures 5A–5E) and all supported myotube formation, with no significant differences detected between the fiber samples. Overall, amongst the fiber samples, the twin-layered C-S PLA:PHA fibers supported longer myotube formation lengths, 98.4 ± 8.8 μm, compared to the C-S PHA:PLA fibers, PLA and PHA blend fibers, 71.7 ± 19.9 μm, 73.1 ± 12.7 μm and 76.3 ± 16.1 μm, respectively. A significant difference was detected between myotubes measured on the TCP control, 108.1 ± 18.4 μm, compared to PLA fibers. The myotube length was found to be highest on the twin-layered C-S PLA: PHA fibers, although all fibers supported C2C12 cell differentiation.

### Human primary osteoblast mineralization

**Figure 6:**
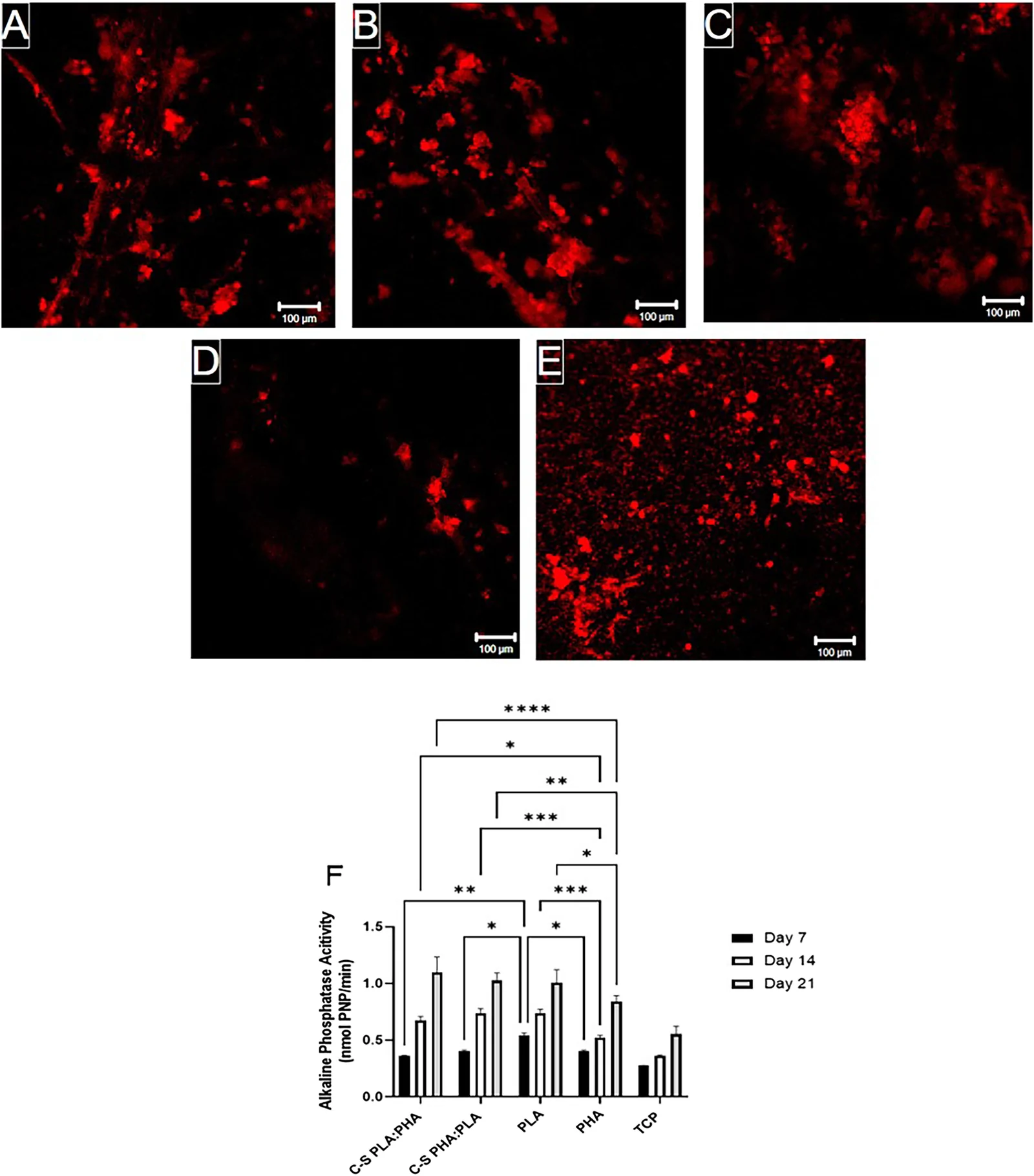
Confocal micrographs of xylenol orange-stained mineralized deposits of human osteoblasts, cultured for 14 days, on (A) C-S PLA: PHA, (B) C-S PHA:PLA, (C) PLA, and (D) PHA blend fibers. (E) Tissue culture plastic control (scale bars, 100 μm): (F) ALP activity of human osteoblasts on samples read at 7, 14 and 21 days (mean ± SD, *n* = 3 independent experiments; ∗*p* < 0.05, ∗∗*p* < 0.01, ∗∗∗*p* < 0.001, and ∗∗∗∗*p* < 0.0001).

Higher amounts of mineralized nodules were observed on the twin-layered C-S fibers (Figures 6A and 6B) and PLA fibers (Figure 6C) compared to the PHA blend fibers (Figure 6D) after culturing human osteoblasts for 14 days. Mineralization was quantified using an alkaline phosphatase (ALP) activity assay at 7, 14, and 21 days (Figure 6F). At 7 days, ALP activity was significantly higher on the mono PLA fibers than the other fiber conditions. However, after 14 days, and 21 days, osteoblast ALP activity was significantly higher on the C-S PLA:PHA, C-S PHA:PLA fibers and PLA fibers compared to the PHA blend fibers and TCP control.

### BRIN-BD11 rat pancreatic cell insulin secretion response

**Figure 7:**
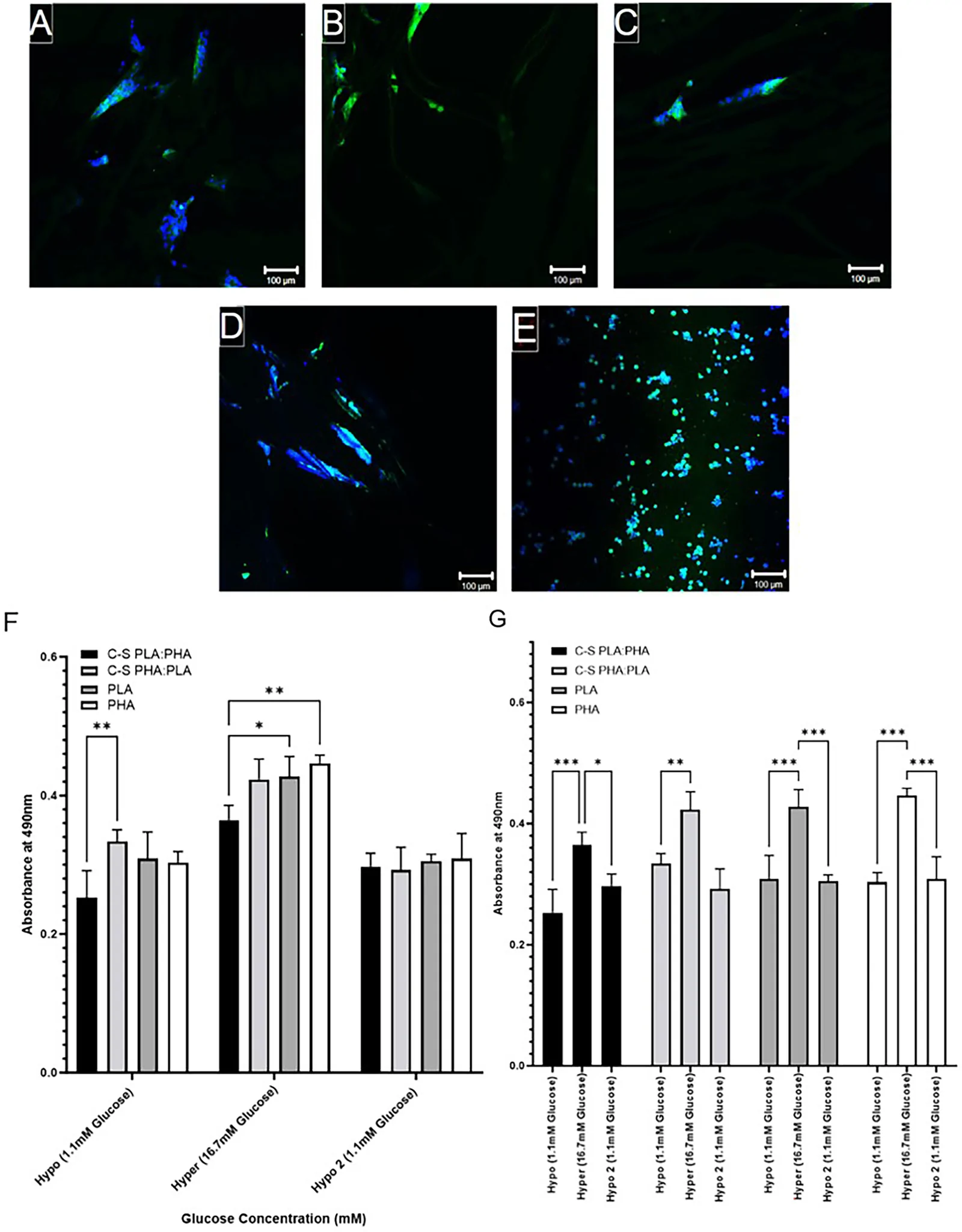
Confocal micrographs of BRIN-BD11 rat pancreatic cells stained for positive insulin staining (green) and DAPI (blue) on (A) C-S PLA: PHA, (B) C-S PHA:PLA, (C) PLA, and (D) PHA fibers. (E) Tissue culture plastic control (scale bars, 100 μm): (F) Effect of alternating glucose concentration in cell media on absorbance of antibody-coupled insulin of BRIN BD11 islet beta cell lines grown on gyro spun fibers and (G) effect of alternating glucose concentration in cell media on absorbance of antibody-coupled insulin of BRIN BD11 islet beta cell lines grown on gyro spun fibers (Mean ± SD, *n* = 3 independent experiments; ∗*p* < 0.05, ∗∗*p* < 0.01, and ∗∗∗*p* < 0.001).

Insulin-positive BRIN-BD11 rat pancreatic beta cell attachment could be visualized on all fiber substrates (Figures 7A–7E) and was quantified using an insulin ELISA assay (Figures 7F and 7G). Cells were exposed to high and low levels of glucose to initiate acute and chronic insulin secretion in response. Significant differences were noted between the alternating low and high glucose concentration conditions in all tested materials. The most significant response was found in the PHA fibers, for which the insulin absorbance measured at hyperglycemia was significantly higher than either hypoglycemic conditions, with an absorbance of 0.45 ± 0.01 compared to 0.30 ± 0.01 and 0.31 ± 0.03 for the 1st and 2nd hypoglycemic groups respectively. All other groups displayed significant rises in detected insulin with elevated glucose concentrations and significant decreases in insulin secretion following a return to 1.1 mM glucose concentrations, with the exception of the C-S PLA:PHA samples which experienced a significant increase of insulin ELISA absorbance following the glucose concentration elevation (0.25 ± 0.04 to 0.36 ± 0.02 respectively), but the decrease in insulin absorbance on return to baseline was not deemed significantly significant. Small significant differences were also observed between each material at each glucose concentration (Figure 7G). C-S PLA:PHA produced significantly less insulin at hypoglycemia 1 compared to C-S PHA:PLA under the first hypoglycemic condition, with the C-S PLA:PHA also performing less well than virgin P(3HB) under hyperglycemic conditions. Upon return to hypoglycemia, no significant differences were found between any of the material groups. All fiber substrates supported insulin secretion from BRIN-BD11 rat pancreatic beta cells under low and high glucose concentrations, as demonstrated by positive insulin staining and detection of insulin secretion using an ELISA assay. Insulin secretion significantly increased between the alternating low and high glucose concentration conditions in all tested materials. However, this was most noticeable on the PHA fibers. The C-S PLA:PHA samples experienced a significant increase in insulin ELISA absorbance following the glucose concentration elevation, but the decrease in insulin absorbance on return to baseline was not deemed significant; therefore, C-S PHA:PLA samples were deemed to be more supportive.

### Collagen IV formation from conditionally immortalized human glomerular podocytes (CIHPs), specialist kidney cells and podocyte viability

**Figure 8:**
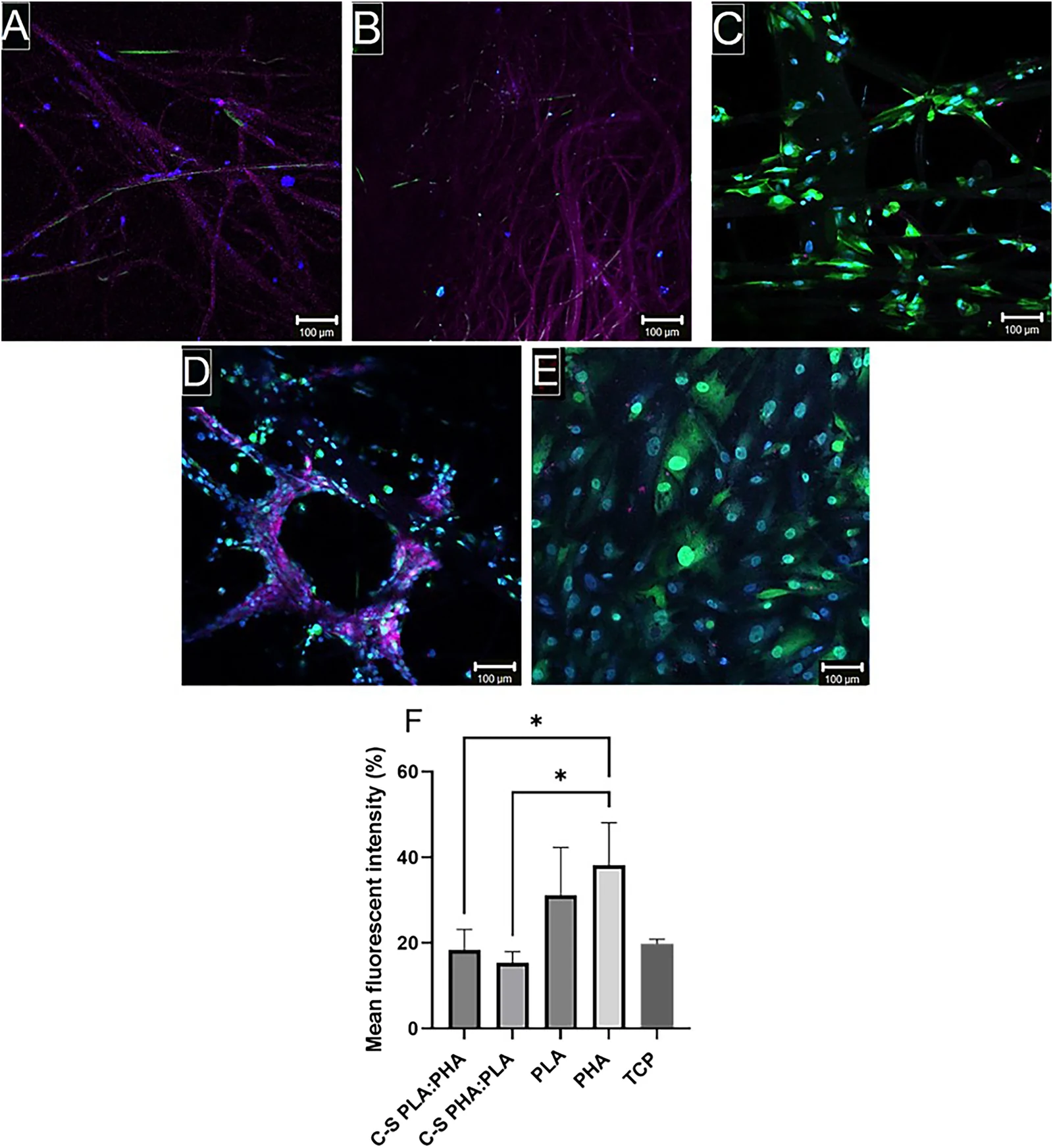
Confocal micrographs illustrating conditionally immortalized human glomerular podocytes (CIHP) stained for DAPI (blue), GFP (green), and collagen IV (purple) cultured on (A) C-S PLA:PHA, (B) C-S PHA:PLA, (C) PLA and (D) PHA fibers. (E) Tissue culture plastic control (scale bars, 100 μm). (F) Mean fluorescent intensity of collagen IV (mean ± SD, *n* = 3 independent experiments ∗*p* < 0.05).

All fiber substrates supported collagen type IV formation from conditionally immortalized human glomerular podocytes (CIHPs) (Figures 8A–8E). Collagen-type IV expression was significantly higher on the PHA blend fibers, 37.1 ± 18.2%, compared to the twin-layered C-S PLA:PHA fibers, 11.4 ± 2.6%, and twin-layered C-S PHA:PLA fibers, 13.3 ± 4.8% (Figure 8F). In some areas, the PHA blend fibers exhibited an overall rounded shape of the CIHPs (Figure 8D), which might have induced the very high collagen production. No other significant differences were detected between datasets. Significantly increased collagen type IV production from glomerular podocytes and endothelial cells was observed on the PHA blend fibers, compared to the other fiber conditions, supporting the formation of kidney-like structures and normal kidney cell phenotype.

## Discussion

In this study, a blend of PHAs, P(3HB)/mcl-PHA 80/20 (PHA blend), was investigated as both the core and sheath of the twin-layered fiber, with PLA, and compared with mono-PHA blend and PLA fibers respectively, investigating production of the fibers, fiber diameter, fiber surface properties and *in vitro* cell responses. We have previously reported on the advantages of PHA blend fibers, manufactured from PG, for both hard and soft tissue engineering applications, but this has not been investigated using the twin-layered C-S fiber technology.^26^^,^^32^ Figure 1 demonstrates the cross-sectional views of C-S PLA:PHA fibers using in-line phase contrast tomography, optical microscopy and fluorescence dyes. The sheath layer of all the fibers was the thinnest compared to the core of the fibers, which was consistent throughout, which is a significant cost advantage of using CS-PG when using expensive polymers as the functional sheath layer or coating for biomedical applications.^5^^,^^8^

Fibers containing the PHA blend, either as the sheath or the mono PHA blend fibers, exhibited increased porosity compared to the PLA sheath and PLA mono fibers. Fibers produced from CS-PG demonstrated no significant difference in fiber size and no changes in ratio between the core and sheath layers in the various fiber types, which could be clearly observed using a range of imaging techniques. Vast quantities of fibers, with similar diameters, were mass produced in a continuous, robust and cost-efficient way using CS-PG, compared to existing technologies, such as coaxial spinning, which is unable to facilitate the production of fibers at this scale for industrial applications. As reported in the literature, coaxial electrospinning has a reduced production rate of 0.054 g h^−1^ compared to core-sheath gyration with a production rate of 225 g h^−1^ (Table S2), as well as the need for large electrical inputs to generate the electric field, which is not required in core-sheath gyration manufacturing methodology.^8^^,^^33^

Pores were observed on all fibers, due to the speed of solvent evaporation of the chloroform in the solution. Mono-PHA blend fibers, and twin-layered PHA blend sheath fibrs, however, exhibited increased porosity, compared to the PLA fibers and the twin-layered PLA sheath fibers. Similar results have been observed in our previous work, when using P(3HB) in PHA blends, in which increased pores form in P(3HB). The addition of P(3HB) to a blend with P(3HO) generated protrusions and pores on the surface, believed to be due to its “rapid crystallization rate and high degree of crystallization.”^32^

The CS-PG technology allowed for the tailoring of fibers with varying mechanical properties, a huge advantage for tissue engineering applications when trying to replicate the ECM of hard tissues, such as bone, and soft tissues such as nerve. As observed in the SEM images, PHA blend fibers, mono and as the fiber sheath, exhibited increased porosity, resulting in increased surface roughness and topography of the fibers. Similar results have been observed in our previous studies, using P(3HB), in a PHA blend with mcl-PHAs, in which Basnett et al. observed an increase in porosity, surface roughness, and protein adsorption when incorporating P(3HB) into a blend with the mcl-PHA P(3HO).^32^ AFM micrographs were used to quantify the surface elastic modulus of the fibers.^34^^,^^35^ Twin-layered C-S fibers with PLA as the core, increased the overall stiffness of the fiber, compared to fibers with a PHA blend core, which was expected. PLA is known to be a stiff polymer, with a Young’s modulus value of 3500 MPa,^36^ whereas PHA blend fibers, in our previous work, P(3HB)/P(3HO-*co*-3HD) 80/20 fibers, had a Young’s modulus of 920 ± 100 MPa.^26^ It was noted that blending P(3HB), with an mcl-PHA, significantly reduced the Young’s modulus and the tensile strength, which has been seen in all our previous studies.^23^^,^^26^ This highlights the advantages of C-S PG to produce fibers with varying mechanical properties and to tailor properties for varying applications in tissue engineering.

The increase in porosity, surface roughness, and protein adsorption of the mono-PHA blend fibers and PHA blend sheath fibers ultimately affected cellular adhesion, viability, and differentiation on all cell types in this study, highlighting the advantages of P(3HB) in the PHA blend. Twin-layered C-S PLA: PHA fibers significantly supported NG108-15 neuronal cell adhesion, demonstrating that the addition of the PHA blend as a sheath to the PLA fibers was highly advantageous in nerve tissue engineering. Our previous studies have highlighted PHA blend fibers, produced via PG and electrospinning, significantly support neuronal cell adhesion, viability and differentiation compared to FDA-approved polymers such as PLA and PCL.^23^^,^^37^^,^^38^ Taylor et al. reported significantly longer neurites growing from NG108-15 neuronal cells, and dorsal root ganglion explants, on P(3HB)/P(3HO) 50/50 electro spun fibers, compared to PCL fibers of the same fiber diameter, due to differences in water contact angle, surface roughness and protein adsorption, favoring the PHAs.^39^ *In vivo*, nerves exhibit layered architectures, for example, the myelin sheath wrapping around nerve fibers.^39^ The C-S fibers can be used to mimic nature’s compartmentalization, by assigning mechanical strength to the PLA core and bioactivity to the PHA sheath.

C2C12 myoblast cells stained positively for myosin on all fiber samples and all supported myotube formation, with no significant differences detected between the fiber samples. These results are consistent with previous findings that show fiber scaffolds support C2C12 myotube formation. However, our results could be significantly improved with the use of more aligned fibers, as seen in studies by Ricotti et al. and Luo et al., who noted significant myotube formation on aligned P(3HB) and PLA scaffolds, respectively, compared to randomly orientated fibers.^40^^,^^41^ The addition of PLA as a core to the twin-layered C-S PLA: PHA fibers, significantly increased viable cell adhesion compared to mono-PHA fibers, which could be responsible for the increase in myotube length formed. Engler et al. observed that C2C12 myoblasts adhered, elongated, and spread more on stiffer substrates than soft substrates, which is in line with our findings, as C-S PLA: PHA fibers exhibited a higher elastic modulus compared to mono-PHA fibers.^42^ *In vivo,* muscle exhibits layered architectures, aligned myofibrils encased within a sarcolemma, forming a core-sheath structure.^43^ The C-S fibers here could be mimicking this, taking direct inspiration from the hierarchical and compartmentalized architecture of muscle tissue. The PLA core provides mechanical and structural integrity, whereby the PHA offers the bioactivity. The setup could be further optimized to replicate the anisotropic microenvironment of muscle by further optimizing fiber diameter, as well as orientation in future work.^44^

A large advantage of CS-PG is the ability to tailor the mechanical properties of the fibers to the tissue type being replicated, in particular in hard tissue engineering. C-S fibers, with PLA as the core, increased overall stiffness of the fibers, which was particularly advantageous when culturing human primary osteoblasts *in vitro*. Both PLA and PHA blends have been investigated previously for bone tissue engineering, with both materials supporting bone regeneration and mineralization, but often with the addition of HA or BMP-4 for bioactivity.^25^^,^^45^ Mono PLA and the twin-layered C-S PLA: PHA fibers, significantly increased adhesion of live viable osteoblasts compared to mono PHA fibers, and the twin-layered C-S PHA: PLA fibers, which exhibited more elastomeric mechanical properties. This was also observed in Abbasi et al., who noted greater osteoblast viability, attachment, and proliferation of osteoblasts on stiffer substrates, compared to softer substrates.^46^ Increased elastic modulus of the mono PLA and the twin-layered C-S PLA:PHA fibers also influenced bone mineralization, as observed with the increased presence of mineralized bone nodules, and ALP activity. Similar results were also observed in Zhang et al., who observed increased osteoblast mineralization, and ALP activity on stiffer substrates with the Young’s modulus value of 120 KPa, compared to softer substrates with Young’s modulus values of 15 and 5 KPa.^47^ Bone is a complex tissue, consisting of a structural hierarchical architecture consisting of a tough collagen matrix reinforced by hydroxyapatite, providing both flexibility and compressive strength.^48^ C-S fibers can emulate the layered architecture of bone, in this case, the PLA core offering mechanical strength, for mimicking load bearing, whereby the PHA sheath provides bioactivity, biocompatibility and controlled degradation.^26^ Furthermore, to improve the bioactivity of the scaffolds, a bioactive reagent can be incorporated into the fiber and released in a controlled manner. For example, Xu et al. incorporated human parathyroid hormone (R-PTH) into a biomimetic micro-structured scaffold, which promoted the reconstruction and regeneration of the injured tendon-to-bone interface.^49^ Xu et al. further investigated the incorporation of Deferoxamine (DFO) into biomimetic scaffolds, which promoted BMP2 and VEGF expression, promoting early and rapid healing of ACL reconstruction *in vivo.*^50^

Insulin-positive BRIN-BD11 rat pancreatic beta cell attachment could be visualized on all fiber substrates. Cells were exposed to high and low levels of glucose to initiate acute and chronic insulin secretion as a response. There is little literature describing how different material types affect BRIN-BD11 insulin production but preliminary studies have shown that P(3HB) and mcl-PHAs do not inhibit BRIN-BD11 insulin release, with similar results observed as with PLA.^30^ More recently, scaffold stiffness has been shown to affect insulin production. Johansen et al. concluded that increased ECM scaffold stiffness, with scaffolds manufactured from Poly(serinol hexamethylene urea)-co-poly(N-isopropylacrylamide) (PSHU-PNIPAAm), inhibited insulin secretion in mouse and human islets, indicating softer scaffolds supported insulin secretion better.^51^ This aligns with our finding in Table 2, in which PHA fibers had the lowest Young’s modulus, of 78.0 ± 2.3 MPa, compared to the PLA fibers and core-sheath fibers, C-S PLA: PHA and C-S PHA: PLA, which had moduli of 178.4 ± 5.7, 213. 3 ± 4.3 and 97. 6 ± 3.6 MPa respectively. Significantly higher numbers of live viable BRIN-BD11 rat pancreatic beta cells were observed on the twin-layered C-S PLA: PHA fibers compared to the other fiber substrates, in their characteristic clustered phenotype, likely due to increased protein adsorption. Previous literature has shown that both PHAs and PLA support BRIN-BD11 rat pancreatic beta cell attachment, growth, and proliferation.^30^^,^^52^ This study also looks at the effect of fibers, including mono-layered and twin-layered fibers, on the insulin production capacity of BRIN-BD11 cells. Naturally, the pancreas is a soft, vascularized organ with delicate endocrine structures (islets of Langerhans).^53^ In terms of biomimicry, the PLA in the C-S PLA: PHA fibers, is acting as a structural backbone, similar to connective tissue in the pancreas. The PHA sheath offers a more biocompatible interface for cell adhesion, as seen in Figure S10. The PHA sheath also offers increased permeability, which, when designing a bioartificial pancreas, is crucial to allow glucose, insulin, and oxygen to permeate through the structure.^54^ Furthermore, PHA as the sheath is beneficial with regard to its degradation profile, degrading into naturally derived metabolites, reducing inflammation, mimicking an immune-privileged environment, crucial for designing biomaterials for pancreatic tissue engineering.^53^^,^^54^

All fiber substrates supported collagen type IV formation from CIHPs. This study investigated PHA fibers in kidney tissue engineering. MCL-PHAs have been investigated for kidney tissue engineering, but in the form of solvent-cast films.^55^^,^^56^ Significantly increased collagen type IV production from glomerular podocytes and endothelial cells was observed on the PHA blend fibers, compared to the other fiber conditions, supporting the formation of kidney-like structures and normal kidney cell phenotype31. Type IV collagen predominantly makes up the glomerular basement membrane, formed by glomerular podocytes and endothelial cells *in vivo.*^51^^,^^53^^,^^54^ Our study suggests that the PHA-blend fibers supported the best collagen IV production.

Previous studies have shown that electro spun fibers support collagen IV production from conditionally immortalized Human Glomerular Podocytes and endothelial cells. Tuffin et al. demonstrated CIHP culture with a poly(glycolic acid) electro spun scaffold, filled with a fibrin hydrogel occupying the interstitial spaces of the scaffold.^55^^,^^56^ Scaffolds supported the proliferation of CIHP cells as well as promoted self-assembly of collagen IV when co-cultured with CiGEnCs.^57^ This work supported the finding by observing similar behavior of the CIHP culture on the twin-layered C-S fibers, especially mono PHA. In addition, this study indicated that curved fiber structures such as those observed in Figure 8 further enhanced collagen IV production. This is a highly significant finding and would be worth exploring further in the future.

Previous work supports our findings that PHAs support kidney tissue repair. Syed Mohammed et al. confirmed a form of MCL-PHA, produced from *P. mendocina* CH50, in film form, supported both proliferation and differentiation of human podocytes and glomerular endothelial cells, and the production of collagen IV, the main component of the glomerular basement membrane.^58^ Other natural materials, such as silk, significantly improve kidney cell viability compared to synthetic materials such as PLA31. Mou et al. manufactured laminin-functionalized silk fibers that supported differentiation of human pluripotent stem cells into mature podocytes, as evidenced by the upregulation of podocin and nephrin (podocyte-specific markers).^37^ PLA has been investigated using kidney cells, in which Burton et al. noted porous PLA fibers, 3.71 μm in diameter, best supported kidney cell proliferation compared to smaller less porous fibers.^57^ This result is similar to our study, as the PHA blend fibers exhibited the most porosity, compared to all the fiber conditions. Hence, porosity and fiber orientation seem to be two very important factors governing the growth and maturation of kidney cells. In terms of biomimicry, the PHA fibers are ideal for supporting kidney tissue engineering applications. Fiber structures can mimic the elongated tubular structures of nephrons, in terms of architecture, by alignment, and layering of fibers to support cell polarity.^59^ Kidney tissues are soft and elastic, in which our PHA blend fibers support this due to the 20% content of MCL-PHA in the PHA blend, closely matching the compliance of glomerular and tubular tissues.^26^ Furthermore, PHAs degrade into natural metabolites, reducing inflammation, which the kidney filtration barrier must avoid.^59^ This study confirms that PHAs support kidney cell adhesion and attachment, and that PHAs in our system, can be further modified with ECM proteins, and growth factors to enhance nephron-like function.^31^

Overall, Table 3 consolidates and summarizes the findings of this extensive study using twin layered/single layered fibrous scaffolds produced using pressurized gyro spinning. The C-S architecture of PHA/PLA fibers offers a versatile platform for tailoring mechanical and biochemical properties to meet diverse biomedical requirements. For example, if requiring a stiffer fiber, such as for bone tissue engineering, C-S PLA: PHA fibers can be utilized for this application. However, if a relatively softer material is required, such as in kidney tissue engineering, then the PHA blend can be chosen as the core of the fibers for this specific application. By independently tuning the core and sheath compositions, the system enables precise modulation of stiffness, elasticity, and degradation kinetics for a wide range of tissue engineering applications. This dual-material configuration also supports biochemical adaptability. PHA’s surface erosion yields biocompatible degradation products that are natural metabolites removed from the body. PLA degrades via bulk degradation into acidic byproducts, causing the surrounding tissue to become slightly acidic. This pH change can be advantageous if designing a medical implant that has stimuli-responsiveness to pH changes, exhibiting dynamic functionality. PG has the ability to control fiber diameter, alignment, and porosity, which further enhances cell guidance, proliferation, and tissue integration. Many cell types had a preference for PHA as the sheath component, confirming the outstanding bioactive properties of the PHAs. In addition, this technology also utilizes the addition of a bioactive material, such as PHA, in a thin layer onto a relatively less bioactive material, such as PLA. This not only improved the biocompatibility of the biopolymer used as the core of the fiber, but also is a highly cost-effective process when using expensive bioactive materials as coatings. Collectively, these features position C-S PHA/PLA fibers as a multifunctional scaffold platform capable of addressing the complex demands of regenerative medicine and targeted therapeutics. This study has unraveled an unprecedented toolbox for the generation of fibrous scaffolds to provide optimized bioactive scaffolds for hard and soft tissue engineering applications, providing a hugely promising repertoire of highly bioactive and tailorable future biomaterials.

**Table 3:** A summary of the results obtained in the context of the different cell types studied in this work

Cell type	Cell viability response	Cell phenotype response
NG108-15 neuronal cells	increased cell attachment on C-S PLA: PHA fibers	increased neurite outgrowth on C-S PLA: PHA fibers
C2C12 myoblast cells	increased cell attachment on C-S PLA: PHA fibers	higher myotube formation on C-S PLA: PHA fibers
Human primary osteoblasts	increased cell attachment on PLA and C-S PLA: PHA fibers	increased mineralization and ALP activity on PLA and C-S PLA: PHA fibers
BRIN-BD11 rat pancreatic beta cells	increased cell attachment on C-S PLA: PHA fibers	all substrates supported insulin-positive cells and maintained normal insulin levels
Conditionally immortalized human glomerular podocytes	increased cell attachment on PHA blend fibers	increased collagen IV staining on PLA and PHA blend fibers
Conditionally immortalized glomerular endothelial cells	increased cell attachment on PHA blend fibers	all substrates exhibited good collagen IV staining
HaCaT immortalized human keratinocytes	increased cell attachment on PLA and C-S PLA: PHA fibers	all substrates supported cytokeratin-positive cells

In conclusion, this study highlights the many advantages of the combination of C-S PG and the highly versatile family of natural and sustainable polymers, PHAs, to produce vast quantities of PHA-based fibers in combination with PLA for varying tissue engineering applications. This work has the potential to overcome the limited availability of highly biocompatible sustainable biomedical polymers suitable for optimal tissue regeneration and organ replacement of hard and soft tissue/organ types. C-S PG can be used to manufacture C-S fibers, in which the material as both the core and the sheath within twin-layered fibers can be changed depending on the needs of the type of tissue being regenerated. The use of C-S fibers is advantageous in utilizing a soft or stiff material as the core of the fiber, depending on the application, with the addition of a thin sheath material. This can be advantageous to manufacturers using expensive bioactive components, such as growth factors within the outer coating, limiting the amount required for the application. In addition, PG overcomes the limitations of conventional spinning technologies such as coaxial electrospinning and melt spinning by generating much larger quantities of polymer fibers, in a sustainable way by not requiring high temperatures or large electric fields. Therefore, the twin-layered C-S fibers, manufactured from C-S PG, are functional, cost-efficient, and scalable for many biomedical applications. This work opens the possibility of using a combination of the properties of PHAs and PLA along with PG to produce fiber-based scaffolds for bone, cardiac, pancreas, kidney (glomerulus), and skin tissue engineering. This is an incredible, unprecedented, versatile technology for a combination of biomaterial/processing method with the potential to revolutionize tissue engineering and organ replacement in the near future.

### Limitations of the study

We have highlighted the potential of C-S fibers as a toolbox of fibrous scaffolds for tissue engineering using *in vitro* tests only, and therefore, there is a lack of verification through the use of animal *in vivo* experiments. Future work will investigate the different combinations of fibers in both relevant *ex vivo* and *in vivo* models to further study biocompatibility, integration, vascularization, immune cell responses, and clearance of degradation products, critical for future regulatory approval. Furthermore, functional recovery assessments will be performed, on top of biocompatibility studies, to quantify nerve conduction and muscle re-innervation, re-mineralization and mechanical strength restoration of bone tissue, glucose tolerance, insulin/C-peptide, glycemia tests for glucose-regulated insulin secretion for the pancreas, and GFR, creatinine/urea, urine output/composition tests to observe filtration, reabsorption and secretion in kidney tissue engineering applications. In areas where immortalized cells have been used, further *in vitro* testing will be conducted on primary cells to assess cell functions, such as qPCR and SEM micrographs of cells on the scaffolds. Additionally, further material and scaffold optimization can be achieved by the mechanical tuning of the C-S ratios, to better match tissue type mechanical properties and achieve better biomimicry. Fiber alignment could be further strengthened for nerve and muscle tissue applications, and anisotropic mechanical properties assessed to predict properties in *in vivo* conditions. The addition of surface modifications to the fiber surface, as well as bioactive agents, will strengthen cell adhesion and cell function. Overall, we have highlighted the limitations of the current system, and the future tests needed to overcome these.

## Resource availability

### Lead contact

Requests for further information and resources should be directed to and will be fulfilled by the Lead Contact, Prof. Ipsita Roy (i.roy@sheffield.ac.uk).

### Materials availability

This study did not generate new unique reagents.

### Data and code availability

• This paper does not report original code
• Data have been deposited into the University of Sheffield library and can be accessed via https://doi.org/10.15131/shef.data.31314523.
• Any additional information required can be made available to the lead contact upon reasonable request.

## Acknowledgments

We wish to acknowledge Dr Alice Pyne for use of the Bruker Dimension Icon AFM and the 10.13039/100016128Henry Royce Institute for Advanced Materials, funded through 10.13039/501100000266EPSRC grant nos. EP/R00661X/1, EP/S019367/1, EP/P02470X/1 and EP/P025285/1. I.R. acknowledges funding from the 10.13039/501100000308British Council: Innovative and Collaborative Research Partnerships Grants under Pakistan UK Education Gateway (ICRG-2020), project no: 105:006326/DIISB/007/2021 and the 10.13039/501100000266Engineering and Physical Sciences Research Council (EPSRC) funding, EP/V012126/1, EP/X021440/1 and EP/X026108/1. E.A. and D.A.G. were funded by the ECOFUNCO project, grant agreement no. 837863. S.M.D.S.M. is funded by the Program Pelajar Cemerlang (Excellent Student Program) by the Public Service Department, the Government of Malaysia. The UCL authors are again grateful to 10.13039/501100000266EPSRC for funding pressurized gyration research (grant nos. EP/S016872/1, EP/N034228/1, EP/L023059/1). H.M. thanks the Persia Educational Foundation (Mirzakhani Scholarship) for supporting her PhD studies at 10.13039/501100000765University College London (UCL). H.A. thanks PAAET-Kuwait for supporting his PhD studies at UCL. We wish to acknowledge the Wolfson Light Microscope Facility at the University of Sheffield for use of the Nikon A1 confocal microscopy. We acknowledge Diamond Light Source for time on Beamline/Lab I-13-2 under Proposal MG33034. Nicholas T. Farr was funded by 10.13039/501100000266EPSRC (EP/V012126/1). We wish to acknowledge Prof. Cornelia Rodenburg for her support and guidance.

## Author contributions

D.A.G., E.A., A.F., S.M.D.S.M., and J.H. manufactured and characterized the P(3HB) and mcl-PHA used in this study, led by D.A.G., A.F., and I.R. H.M. manufactured and characterized the C-S fibers from pressurized gyration, the production rate, fiber diameter and ratio, SEM and cross-sectional imaging. D.A.G. and N.T.F. performed In-line phase contrast tomography at the Diamond Light Source. D.A.G. performed SEM and AFM analysis of the fibers. C.S.T. performed water contact angle analysis and protein adsorption studies. C.S.T. led *in vitro* cell studies on the fibers. E.A. conducted NG108-15 neuronal cell culture, and C.S.T. conducted imaging and data analysis. A.F. conducted C2C12 myoblast cell culture, and C.S.T. conducted imaging and data analysis. C.S.T. conducted human primary osteoblasts and HACAT cell culture, imaging, and data analysis. J.H. conducted BRIND-B11 cell culture and data analysis, and C.S.T. conducted imaging. S.M.D.S.M. conducted CHIP and CiGenC cell culture, imaging and data analysis. H.M. wrote the introduction, methods, results, and discussion parts for C-S fibers. C.S.T. wrote the introduction, methods, results, and discussion for the *in vitro* cell culture parts. D.A.G., S.M.D.S.M., and A.F. contributed to sections of the manuscript; A.F.M. and S.E.M. renal cell and PHA production, respectively. H.A. oversaw and advised on the production of the fibers. G.I.W. oversaw and advised on kidney cell culture. M.E. and I.R. mobilized funds, conceived the idea, and edited the manuscript. All authors have read and approved the submitted version.

## Declaration of interests

Prof. I. Roy (CSO) and Dr. D. A. Gregory (Technical Advisor) are Directors of PHAsT Limited. Dr. Syed Mohammad Daniel Syed Mohamed is the Analytical Chemist in PHAsT Limited. PHAsT Limited develops biomedical grade polyhydroxyalkanoates (PHAs) for a variety of applications including healthcare and biomedical applications. The polymer used in this work was, however, produced in the Roy Lab.

## STAR★Methods

### Key resources table

**Table:** 

REAGENT or RESOURCE	SOURCE	IDENTIFIER
**Antibodies**		

Mouse monoclonal anti-β III-tubulin antibody	Promega	RRID: AB_430874
Mouse monoclonal anti-myosin antibody	Abcam	RRID: AB_2921304
Rabbit polyclonal anti-insulin antibody	Abcam	RRID: AB_1925116
Rabbit polyclonal anti-keratin 10 antibody	Abcam	RRID: AB_1523465
Texas Red-conjugated Horse anti-mouse IgG antibody	Vector Labs	RRID: AB_2336178
Goat anti-Rabbit Alexa 488 conjugated antibody	ThermoFisher	RRID: AB_143165
Collagen IV mouse polyclonal	eBioscience	RRID: AB_2574403

**Bacterial and virus strains**		

*Pseudomonas mendocina* CH50	University of Sheffield	N/A
*Bacillus subtilis* OK2	University of Sheffield	N/A

**Experimental models: Cell lines**		

NG108-15 neuronal cells	ECACC	RRID:CVCL_0464
C2C12 myoblast cells	ATCC	RRID:CVCL_0188
Human Primary Osteoblasts	Promocell	Cat. No. C-12720
BRIN-BD11 rat pancreatic beta cells	ECACC	RRID:CVCL_6811
HaCaT immortalised human keratinocytes	AddexBio	RRID: CVCL_0038
Conditionally immortalized human podocytes	University of Bristol	N/A
Conditionally immortalized human glomerular endothelial cells	University of Bristol	N/A

**Chemicals, pepties and recombinant proteins**		

Polylactic acid	Merck	38534
Chloroform	Fisher Scientific	CAS: 67-66-3
Xylenol orange	Merck	398187

**Critical commercial assays**		

Propidium iodide	Merck	P4864
Syto 9	Thermofisher	S34854
Rat/Mouse Insulin ELISA 96 well plate kit	Merck Millipore	EZRMI-13BK
Pierce™ pNPP Substrate Kit	Thermo Scientific	37620

**Other**		

Osteoblast Growth Medium (ready to use)	Promocell	C-27001
Osteoblast Mineralization Medium	Promocell	C-27020

### Experimental model and study participant details

#### Microbe strains

Pseudomonas mendocina CH50 (from the University of Sheffield bank) and Bacillus subtilis OK2 (from the University of Sheffield bank). Sex is unknown. A single colony of Bacillus subtilis OK2 was used to inoculate sterile nutrient broth and incubated for 16 h at 30 °C and 200 rpm 26. The seed culture was used to inoculate modified Kannan and Reface media (P3HB production media) in a 15 L Applikon bioreactor, and cultured for 48 h, 30 °C and 200 rpm.^26^^,^^60^ A single colony of Pseudomonas mendocina CH50 was used to inoculate sterile nutrient broth and incubated for 16 h at 30 °C and 200 rpm, before inoculating a second seed culture, in mineral salt medium, for a further 16 h at 30 °C and 200 rpm, using a Brunswick Innova 4430 Incubator Shaker, GMI Inc. Once the cells entered mid-log phase, they were then used to inoculate the final production mineral salt media in a 15 L sterilise Applikon bioreactor, and cultured for 48 h, 30 °C, 200 rpm and an airflow rate of 1 vvm.^26^^,^^61^

#### Cell lines

NG108-15 (ECACC 88112303 at passage 16), C2C12 myoblast cells (ATCC, CRL-1772 at passage 19) and HaCaTs (AddexBio - T0020001, passage 7) were cultured in Dulbecco’s modified Eagle medium (DMEM) supplemented with 10% (v/v) foetal calf serum, 1% (w/v) glutamine, 1% (w/v) penicillin/streptomycin, and 0.25% (w/v) amphotericin B at 37°C and 5% CO_2_ prior and during experimental culture. T-75 flasks containing cells were trypsinized prior to experiments when 80-90% confluent59. BRIN-BD11 rat pancreatic beta cells (ECACC 10033003 at passage 83) were cultured in RPMI-1640 media as previously described.^52^ Human primary osteoblast cells (HoBs) (Promocell.) were cultured in Osteoblast growth medium (Promocell) in a humidified atmosphere with 5% CO_2_ at 37°C. Cells were passaged every 5-7 days when 80% confluent. Cells were used at passage 4 for experiments.^62^ Sex of the cells is unknown and cell lines have been authenticated through purchase. To initiate NG108-15 neuronal cell neurite outgrowth, and C2C12 myotube formation, cell culture medium, containing 10% FCS was swapped to serum-free containing media after 2 days in culture as previously described59. Cells were left for a further 5 days prior to staining and imaging. Conditionally Immortalized human glomerular podocytes were manufactured and provided by The University of Bristol. Conditionally immortalized human podocytes (CIHP) were cultured in RPMI-1640 (Product no. R8758) media with 1% v/v Insulin-Transferrin-Selenium (ITS) as previously described.^63^ Cells were used at passage 20 for experiments. Sex of the cells is unknown and cell lines have been authenticated through purchase. Conditionally Immortalized Glomerular Endothelial cells were provided by The University of Bristol. CiGEnCs were manufactured and cultured in EGM2-MV BulletKit (Product no. CC-3156 & CC-4147) as previously described.^64^ Cells were used at passage 20 for experiments. Sex of the cells is unknown and cell lines have been authenticated through purchase.

### Method details

#### Production of P(3HB)

Production of P(3HB) was performed with *Bacillus subtilis* OK2 and extracted then purified as previously described.^26^ Briefly, a single colony of *Bacillus subtilis* OK2 was used to inoculate sterile nutrient broth and incubated for 16 h at 30 °C and 200 rpm.^26^ The seed culture was used to inoculate modified Kannan and Reface media (P3HB production media) in a 15 L Applikon bioreactor, and cultured for 48 h, 30 °C and 200 rpm.^26^^,^^65^ Purification was performed using the soxhlet method, and characterization performed as previously described and included in supplementary information.^60^

#### Production of MCL-PHA

MCL-PHA production was performed with *Pseudomonas mendocina* CH50, and extracted then purified as previously described.^26^ Briefly, a single colony of *Pseudomonas mendocina* CH50 was used to inoculate sterile nutrient broth and incubated for 16 h at 30 °C and 200 rpm, before inoculating a second seed culture, in mineral salt medium, for a further 16 h at 30 °C and 200 rpm, using a Brunswick Innova 4430 Incubator Shaker, GMI Inc. Once the cells entered mid-log phase, they were then used to inoculate the final production mineral salt media in a 15 L sterilise Applikon bioreactor, and cultured for 48 h, 30 °C, 200 rpm and an airflow rate of 1 vvm.^26^^,^^60^^,^^61^ Purification was performed using the soxhlet method, and characterization performed as previously described and included in supplementary information.^26^

#### Characterization of Polyhydroxyalkanoates

Characterization of Polyhydroxyalkanoates was performed as per the methods in Basnett et al. in full.^26^

##### Fourier transform infrared spectroscopy (FTIR)

FTIR measurements were carried out using this. Absorbance spectra were registered between 400 and 4000 cm−1, with a resolution of 4 cm−1 and 45 scans.^26^

##### Nuclear Magnetic Resonance (NMR)

Two types of Nuclear Magnetic Resonance (NMR) spectroscopy were performed: 1H NMR (proton NMR) and 13C NMR (carbon NMR). Both analyses were conducted using a Bruker AVI 400 MHz spectrometer for solution-state samples. The polymer samples were dissolved in deuterated chloroform, with tetramethylsilane (TMS) serving as the internal reference, detected at 0 ppm. For 1H NMR, spectra were acquired at a frequency of 400 MHz. In the case of 13C NMR, signals were recorded at 100 MHz, for both scl- and mcl-PHA.^26^

##### Differential Scanning Calorimetry (DSC)

The glass transition temperature (Tg) and melting temperature (Tm) of the polymer samples were determined using Differential Scanning Calorimetry (DSC). Each polymer sample was weighed and sealed in a DSC aluminum heating pan. The analysis was carried out using the PerkinElmer DSC4000 instrument, following a two-cycle heating and cooling protocol. The protocol involved heating the sample from -80°C to 200°C at a rate of 10 °C/min, maintaining an isothermal condition at 200 °C for 5 minutes, and then cooling it back to -80 °C at the same rate. This heating and cooling process was repeated once, ending at 30 °C. Prior to analyzing the sample, an empty heating pan was run to establish a baseline. This baseline data was subtracted from the sample's results to obtain a clean and accurate DSC curve.^26^

##### Molecular weight determination of produced PHAs

The molecular weight of the polymer was studied using GPC (1260 Infinity II GPC/SEC system, Agilent, UK) equipped with PL-gel 5μm Mixed-C. The data was analyzed using “Cirrus GPC Version 1.11” software as per previously described.^26^

#### Production of core-sheath fibers

C-S PG device was used for the experiments carried out in production of C-S PLA: PHA, C-S PHA:PLA, PLA and PHA fibers. The device consists of a hexagonal shaped cabinet, dual cylindrical reservoir, containing a core chamber placed at the centre of the sheath vessel with 60 mm outer diameter and 13mm inner diameter. There are 4 concentric orifices with a 2.2 mm outer diameter and 1.25 mm inner diameter located at the exact centre of the pot. Furthermore, the top of the vessel is attached to a pressurized gas (N2) that can provide 0.1 – 0.3 MPa flow pressure and the bottom of the vessel is connected to a high-speed DC motor capable of providing up to 12,000 rpm.^8^

**Figure:**
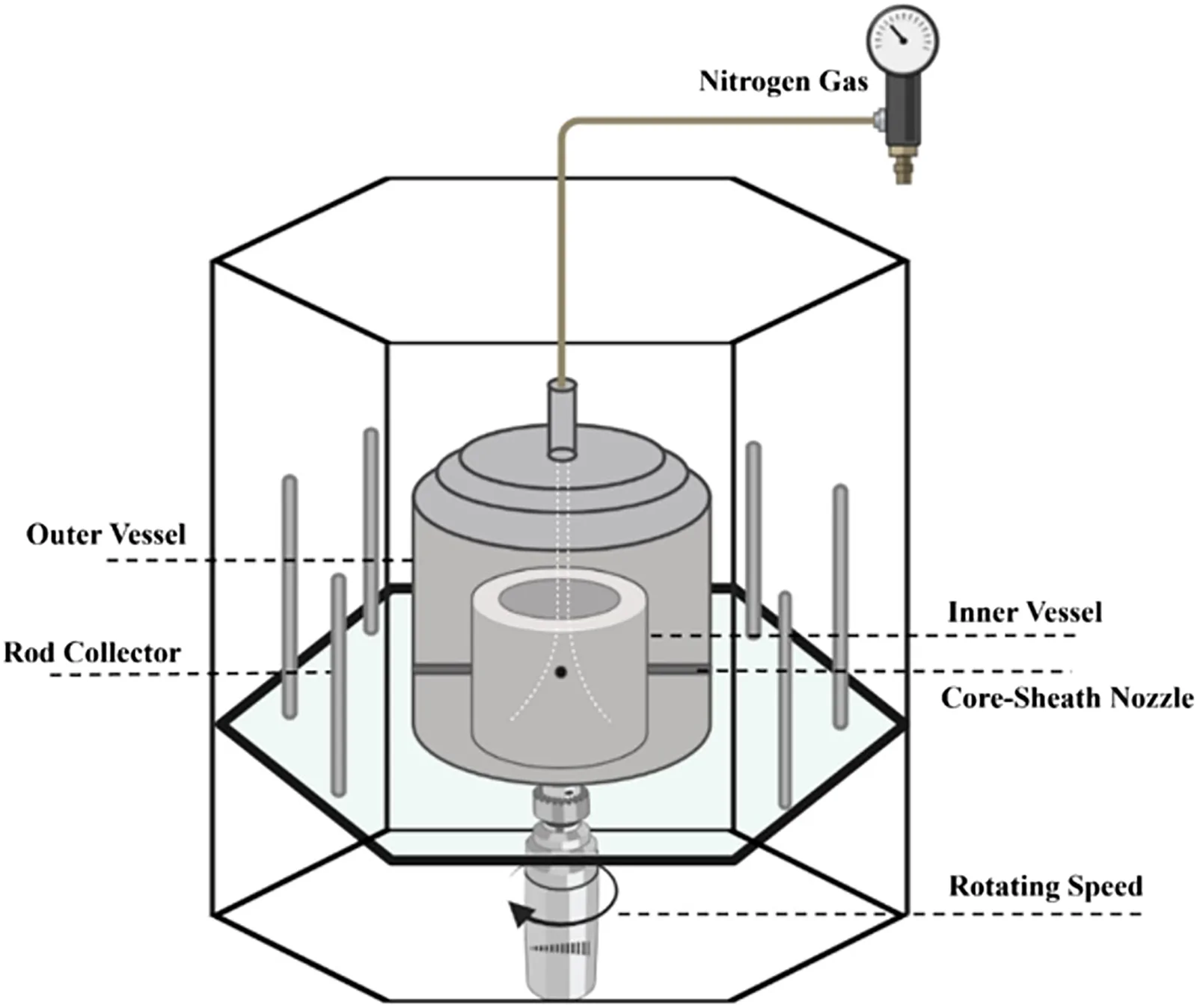
Schematic of the core-sheath pressurised gyration set up

##### Core-sheath PHA

***PLA – PLA:PHA Fibers:*** 3 mL of 20 % (w/v) PLA polymer solution, was loaded into the inner vessel to act as the core of the fiber and 3 mL volume of 11 % (w/v) P(3HB)/mcl-PHA polymer solution in the outer vessel to act as the sheath of the fiber. Solutions were spun for 15 s at a fixed pressure of 0.1 MPa and a fixed rotating speed of 6,000 rpm under ambient conditions (19.9 °C and 47- 52 % relative humidity) to form fibrous scaffolds.^4^

***PLA fibers***: 3 mL volume of 20 % (w/v) PLA, in chloroform, was loaded in the inner vessel of the gyrator device and the outer vessel of the gyrator was left empty. The solution was spun for 15 s at a fixed pressure of 0.1 MPa and a constant rotational speed of 6,000 rpm under ambient conditions (19.9 °C and 48-55 % relative humidity) to form fibrous scaffolds^4^

***PHA fibers***: 3 mL volume of 11 % (w/v) P(3HB)/MCL-PHA, in chloroform, was loaded in the inner vessel of the gyrator device and the outer vessel of the gyrator was left empty. The solution was spun for 15 s at a fixed pressure of 0.1 MPa and a constant rotational speed of 6,000 rpm under ambient conditions (19.9 °C and 48-55 % relative humidity) to form fibrous scaffolds. The experiments were performed in triplicate.^4^

#### Characterization of cross-sections of core-sheath fibers

Cross-sectional characterization of the core-sheath fibers was performed via in-line phase contrast tomography, scanning electron microscopy and optical microscopy. In-line phase contrast imaging and tomography was performed on the 13-2 Diamond Manchester Imaging Branchline at the Diamond Light Source UK. A micro-imaging set up for samples with 0.1-10 mm thickness was selected. The high flux of a synchrotron enabled images of high signal:noise to be recorded. For all fibers a 0 – 180-degree scan was applied with a 0.006 step size. 0.5 seconds exposure was selected. 10 x objective was selected for all the measurements. After 3D reconstruction was completed using an in-house MATLAB package, 3D image analysis was performed using Avizo Software (Thermo Fisher Scientific). To obtain cross-section images of the C-S PLA: PHA fibers and analyze the ratio between the diameter of the core and the sheath, an EVO 10 (SEM) (Zesis, Germany) at an accelerating voltage of 3 kV was used. For this characterization specifically, a lower rotational speed of 2,000 and 4,000 rpm with a constant pressure of (0.1 MPa) was applied during the spinning process to produce coarser fibers with a broad range of 1-15 μm diameter. This was done to prevent damaging the surface cross-section during the cutting stage of the fibers since fibers with a smaller diameter tend to smudge once the blade slides through the strands. Furthermore, the sample preparation involved slicing the fibers using a sharp (SURGICLASS) surgical scalpel (No.11) to achieve a precise cut. The core and sheath structure of the fibers were analyzed using an optical microscopy (VHX-7000 Digital microscope) (Keyence, Japan). To differentiate between the core and the sheath layer, a fluorescent dye rhodamine B (excitation at 543 nm) was dissolved at a concentration of 1 wt % into the respective PHA solution for the sheath of the fibers. The produced C-S PLA: PHA fibers were collected on a glass slide for imaging and processed using Image J software. Fibers at different diameters were selected to analyze the ratio between the diameter of the core and the sheath.

#### Characterization of core-sheath fibers by scanning electron microscopy

The surface morphology of the fibers was analyzed using a scanning electron microscope (SEM) (Hitachi S-3400n) at an accelerating voltage of 5 kV. The surface of the samples was gold sputter-coated for 90 s using (Q150R ES, Quorum Technologies). The micrographs obtained from SEM imaging were analyzed using Image J software to calculate the average fiber diameter and size distribution. For this study, 100 strands of fibers from each study were selected at random for size measurements. The average fiber diameter was calculated and plotted using Origin Pro software. A comparative analysis between four samples was carried out using the fiber diameter and surface morphology data.

#### Characterization by water contact angle measurements

Water contact angle measurements were carried out using the sessile drop method, on an Attension Theta Lite Optical Tensiometer (TL100, Attension, Biolin Scientific, Finland)32. Three technical repeats were used for each measurement, with 10 replicate measurements taken across the surface of 2 x cover slips (5 measurements from each). Infra-red images of the droplets were recorded at 15 fps for 10 s and analyzed using One-Attension software.

#### Characterization of core-sheath fibers by atomic force microscopy

AFM microscopy was performed in a tapping model with SCANASYST-AIR probes under ambient conditions on a Bruker Dimension Icon AFM. Core-sheath fibers were cast onto cover glasses and then stuck to a magnetic AFM support. Different areas of the samples were then analyzed and characterized in Bruckers NanoScope Analysis software (Version 2.0). The data was further averaged by weight dependence on the surface area measured to obtain accurate roughness *Rq* (Root Mean Squared Roughness) and *Ra* (Roughness Average) and adhesion values (how adhesive the polymer fiber is to the tip of the AFM cantilever). Mechanical analysis was performed by fitting data with the Derjaguin–Muller–Toporov (DMT) model to determine the elastic modulus of modified and unmodified fibers, as previously described.^39^

#### Protein adsorption assay

A protein adsorption assay was performed on fiber scaffolds to determine the quantity of adsorbed proteins, from undiluted foetal bovine serum (FBS), as previously described32. Briefly, fiber scaffolds were cut into 10mm x 10mm squares and incubated with 400 μL of undiluted foetal bovine serum (FBS) at 37 °C for 24 h. Samples were washed once with PBS, removed and placed into a new well plate. 1 mL of 2% sodium dodecyl sulphate (SDS), in PBS, was added to each scaffold for 24 h at ambient temperature, under shaking conditions. A Bicinchoninic acid assay was then performed to measure absorbance at 562 nm.^32^

#### Preparation of core-sheath fibers for *in vitro* cell culture

After manufacture, core-sheath and mono polymer fibers were cut into 20 mm x 20 mm sections, to fit into cell culture plates. Fiber sections were ‘glued’ into place, in well plates, using 2.5 % agarose in diH_2_O. To minimize staining surface area, if using a 6 well plate, fibers were held in place using medical grade 316 stainless steel cell culture rings as previously described.^66^ Samples were sterilised with 70% ethanol for 1 h and washed three times with PBS, once overnight, prior to cell seeding. For each sample, 10,000 cells were seeded onto scaffolds, and left in culture for 7- 21 days, depending on the experiment.^66^

#### Immunolabeling of cell types on core-sheath scaffolds

Cells cultured on the fiber scaffolds were labelled for cell type specific antigens, to identify cell type and perform quantitative analysis. For all cell types, culture medium was removed from samples, samples washed with PBS and cells fixed with 3.7% paraformaldehyde for 20 min and permeabilized with 0.1% Triton X-100 for 40 min. NG108-15 neuronal cells, C2C12 myoblast cells, BRIN-BD11 cells and HaCaT cells were stained for specific cell type antigens using a Mouse monoclonal anti-β III-tubulin antibody, Mouse monoclonal anti-myosin antibody, Rabbit polyclonal anti-insulin antibody and Rabbit polyclonal anti-keratin 10 antibody respectively, (1:500 dilution) for 24 h at 4°C, followed by a wash in PBS. Cells were then labeled with a secondary label conjugated antibody (1:250) for 3h at ambient temperature. To visualise cells, a Texas Red-conjugated Horse anti-mouse IgG antibody was used, to visualise NG108-15 neuronal cells and C2C12 myoblast cells. A Goat anti-Rabbit Alexa 488 conjugated antibody was used as a secondary antibody to visualise BRIN-BD11 rat pancreatic beta cells and HaCaT immortalized human keratinocytes. Cells were then labeled with 4’,6-diamidino-2-phenylindole (DAPI, 300 nM, Sigma), to visualise cell nuclei, for 1 h at ambient temperature. The production of collagen IV, from co-culture models of CIHPs and CiGEnCs was evaluated by staining using a Mouse monoclonal collagen IV antibody. CIHPs were transfected to overexpressing stable GFP-actin and mCherry-actin for CiGEnCs as previously reported54, 55. Cells were immersed in PBS and visualised using an upright Zeiss LSM 510 confocal microscope or a Nikon A1 inverted confocal.

#### Human osteoblast mineralisation assay

Mineralization of human primary osteoblasts, on samples, was determined using the methods as previously described to visualise mineralized nodules.^62^ Briefly, Osteoblasts were seeded onto fibers and cultured in human osteoblast growth medium for 24 h. After 24 h culture, medium was replaced with Osteoblast differentiation medium and cells cultured for 13 days. Medium was then replaced with fresh medium containing 0.001% (w/v) xylenol orange, which was incubated overnight to stain mineralized nodules. Medium was removed and samples washed in PBS prior to imaging with a Zeiss LSM 510 META upright confocal microscope.^62^

#### Human osteoblast alkaline phosphatase activity

Alkaline phosphatase activity of human primary osteoblasts, cultured on samples, was determined using the methods as previously described.^67^ Briefly, media was removed and 500 μL of cell digestion buffer (10 v/v% cell assay buffer (1.5 M Tris–HCl, 1 mM ZnCl2, 1 mM MgCl2 in deionised water (diH2O), 1% Triton-X100 (Sigma Aldrich, UK), in diH2O) added to scaffolds and incubated at ambient temperature for 30 min. The lysate was removed and added to 1.5 mL tubes, vortexed and stored at 4 °C overnight.^68^ Lysates underwent a freeze-thaw cycle three times (−80 °C 10 min, 37 °C 15 min) prior to centrifuging samples at 10,000 rpm for 5 min43. A Pierce™ pNPP Substrate Kit (Thermo Scientific) was used to quantify ALP activity. 10 μl of the lysate was combined with 190 μl of PNPP Phosphatase Substrate, added to a 96 well plate and incubated at 37 °C to observe a color change. A BIO-TEK ELx800 plate reader was used to read absorbance at 405 nm, every minute for 30 min44. ‘ALP activity is expressed as nmol of *p*-nitrophenol per minute (nmol pNP/min), assuming that one absorbance value equals 22.5 nmol of product’.^67^

#### BRIN-BD11 rat pancreatic beta cell insulin secretion

BRIN-BD11 cells were cultured onto scaffolds, and insulin secretion determined as per methods previously described, observing acute and chronic secretion rates45. Briefly, acute and chronic insulin secretion was initiated after incubating the cells for 40 min in KRB pH 7.4 containing 1.1 mM glucose, and KRB pH 7.4 containing 16.7 mM glucose plus 10 mM alanine respectively. Insulin secretion was determined using a Rat/Mouse Insulin ELISA 96 well plate kit (Merck Millipore.).^69^

#### Live/dead analysis of core-sheath fiber scaffolds

Samples were stained with 0.001% Syto-9 (Invitrogen) and 0.0015% propidium iodide (Invitrogen) at 37 °C for 30 min 6. Samples were imaged using an upright Zeiss LSM 510 confocal microscope. Three fields of view were taken for each sample, and cells were counted using the ITCN cell counter software on ImageJ (threshold = 40 to 90). The average number of live cells versus dead cells ± standard deviation (SD) per sample were plotted on a graph as well as expressed as a percentage of cell viability.^70^

### Quantification and statistical analysis

GraphPad Instat (GraphPad Software, USA) was used to perform statistical analysis. One-way analysis of variance (ANOVA; *p* <0.05) was conducted to analyze the differences between data sets, incorporating Tukey’s multiple comparisons test if *p* < 0.05. Two-way analysis of variance (*p* < 0.05) was conducted to analyze the differences between data sets when assessing live/dead cell numbers, incorporating a Sidaks multiple comparisons test if *p* < 0.05. The data were reported as mean SD, *p* <0.05, *n*=3 in the figure legend. Each experiment was performed three independent times with each sample repeated three times as *n*=3, unless otherwise stated differently.
