# Supplementary material for: Versatile twin layer macromolecular fibers for advanced tissue engineering applications
Source: iScience. 2026 Sep 10;29(9):116480. doi: 10.1016/j.isci.2026.116480 (PMC13586692; doi:10.1016/j.isci.2026.116480)
Supplement: Supplementary file 1 [file mmc1.pdf]

iScience, Volume *29*

Supplemental information

Versatile twin layer macromolecular fibers for advanced tissue engineering applications

Caroline S. Taylor, Hamta Majd, David A. Gregory, Annabelle Fricker, Syed Mohammad Daniel Syed Mohamed, Jonathan Hinchcliffe, Emmanuel Asare, Nicholas T. Farr, Hussain Alenezi, Gavin I. Welsh, Mohan Edirisinghe, and Ipsita Roy

Versatile twin layer macromolecular fibres for advanced tissue engineering applications

Caroline S. Taylor[1,2*], Hamta Majd[3*], David A. Gregory[1,4,], Annabelle Fricker[1], Syed Mohammad Daniel Syed Mohamed[1,5], Jonathan Hinchcliffe[1], Emmanuel Asare[1], Nicholas T. Farr[1], Hussain Alenezi[3,6], Gavin I. Welsh[5], Mohan Edirisinghe[3], Ipsita Roy[1,4]**

[1] School of Chemical, Materials and Biological Engineering, The University of Sheffield, Sheffield, S3 7HQ, UK

[2] Institute of Medical and Biological Engineering, School of Biomedical Sciences, Faculty of Biological Sciences, University of Leeds, Leeds, UK

[3] Department of Mechanical Engineering, University College London, London WC1E 7JE, UK

[4] Insigneo Institute of In Silico Medicine, University of Sheffield, Sheffield, UK

[5] Bristol Renal, Translational Health Sciences, Bristol Medical School, University of Bristol, Bristol, BS1 3NY, UK

[6] Department of Manufacturing Engineering, College of Technological Studies, PAAET, 13092 Kuwait City, Kuwait

S2 Experimental Section

S2.1 Characterisation of Polyhydroxyalkanoates

Characterisation of Polyhydroxyalkanoates was performed as per the methods in Basnett *et al.* in full [1].

Fourier transform infrared spectroscopy (FTIR): FTIR measurements were carried out using this. Absorbance spectra were registered between 400 and 4000 cm−1, with a resolution of 4 cm−1 and 45 scans [2].

Nuclear Magnetic Resonance (NMR)**:** Two types of Nuclear Magnetic Resonance (NMR) spectroscopy were performed: 1H NMR (proton NMR) and 13C NMR (carbon NMR). Both analyses were conducted using a Bruker AVI 400 MHz spectrometer for solution-state samples. The polymer samples were dissolved in deuterated chloroform, with tetramethylsilane (TMS) serving as the internal reference, detected at 0 ppm. For 1H NMR, spectra were acquired at a frequency of 400 MHz. In the case of 13C NMR, signals were recorded at 100 MHz, for both scl- and mcl-PHA [1].

Differential Scanning Calorimetry (DSC)**:** The glass transition temperature (Tg) and melting temperature (Tm) of the polymer samples were determined using Differential Scanning Calorimetry (DSC). Each polymer sample was weighed and sealed in a DSC aluminium heating pan. The analysis was carried out using the PerkinElmer DSC4000 instrument,

following a two-cycle heating and cooling protocol. The protocol involved heating the sample from -80 °C to 200 °C at a rate of 10 °C/min, maintaining an isothermal condition at 200 °C for 5 minutes, and then cooling it back to -80 °C at the same rate. This heating and cooling process was repeated once, ending at 30 °C. Prior to analysing the sample, an empty heating pan was run to establish a baseline. This baseline data was subtracted from the sample's results to obtain a clean and accurate DSC curve [1].

Molecular weight determination of produced PHAs: The molecular weight of the polymer was studied using GPC (1260 Infinity II GPC/SEC system, Agilent, UK) equipped with PL-gel 5µm Mixed-C. The data was analysed using "Cirrus GPC Version 1.11" software as per previously described [3].

S2.2 Production of Core-Sheath Fibres

Core-Sheath PHA: PLA – PLA:PHA Fibres: 3 mL of 20 % (w/v) PLA polymer solution, was loaded into the inner vessel to act as the core of the fibre and 3 mL volume of 11 % (w/v) P(3HB)/mcl-PHA polymer solution in the outer vessel to act as the sheath of the fibre. Solutions were spun for 15 s at a fixed pressure of 0.1 MPa and a fixed rotating speed of 6,000 rpm under ambient conditions (19.9 °C and 47- 52 % relative humidity) to form fibrous scaffolds [4].

PLA Fibres: 3 mL volume of 20 % (w/v) PLA, in chloroform, was loaded in the inner vessel of the gyrator device and the outer vessel of the gyrator was left empty. The solution was spun for 15 s at a fixed pressure of 0.1 MPa and a constant rotational speed of 6,000 rpm under ambient conditions (19.9 °C and 48-55 % relative humidity) to form fibrous scaffolds [4]

PHA Fibres: 3 mL volume of 11 % (w/v) P(3HB)/MCL-PHA, in chloroform, was loaded in the inner vessel of the gyrator device and the outer vessel of the gyrator was left empty. The solution was spun for 15 s at a fixed pressure of 0.1 MPa and a constant rotational speed of 6,000 rpm under ambient conditions (19.9 °C and 48-55 % relative humidity) to form fibrous scaffolds. The experiments were performed in triplicate [4].

S2.3 Fibre Production Rate

The production yield of PHA, PLA, C-S PLA: PHA and C-S PHA:PLA fibres fabricated by the C-S PG has been analysed by measuring the fibre mass generated using a (Kern ADB Analytical Balance) and calculating the time taken to spin using a (Brannan LCD Display Triple Timer). It should be emphasised that the production rate of C-S PG reported in this work only represents data for the chosen polymers of this project and the production rate will vary

depending on the polymer, solvent, ambient parameters, and operating process.

S2.4 Fibre Solution Rheology

The viscosity and surface tension of the polymer solutions were characterised using a calibrated Kruss tensiometer (Kruss GmbH, Hamburg, Germany) with a standard Du Nouy ring method and a Brookfield viscosity meter (AMETEK Brookfield, Harlow, UK) with an SCV-18 spindle attached. The tests were repeated three times at an ambient temperature of approximately 23 °C (Table S1).

Table S1. Physical properties of PHA and PLA polymer solutions.

**Polymer**	**Concentration % (w/v)**	**Solvent**	**Surface Tension ($mN\ m^{-1}$)**	**Viscosity (mPa s)**
**PHA**	11	Chloroform	49.1 ± 0.9	1252.7 ± 20.8
**PLA**	20	Chloroform	47.3 ± 0.9	2175 ± 16.7

S2.5 Characterisation by Water Contact Angle Measurements

Water contact angle measurements were carried out using the sessile drop method, on an Attension Theta Lite Optical Tensiometer (TL100, Attension, Biolin Scientific, Finland) [1]. Three technical repeats were used for each measurement, with 10 replicate measurements taken across the surface of 2 cover slips (5 measurements from each). Infra-red images of the droplets were recorded at 15 fps for 10 s and analysed using One-Attension software.

S2.6 Protein Adsorption Assay

A protein adsorption assay was performed on fibre scaffolds to determine the quantity of adsorbed proteins, from undiluted foetal bovine serum (FBS), as previously described [5]. Briefly, fibre scaffolds were cut into 10mm x 10mm squares and incubated with 400 µL of undiluted foetal bovine serum (FBS) at 37 °C for 24 h. Samples were washed once with PBS, removed and placed into a new well plate. 1 mL of 2% sodium dodecyl sulphate (SDS), in PBS, was added to each scaffold for 24 h at ambient temperature, under shaking conditions. A Bicinchoninic acid assay was then performed to measure absorbance at 562 nm [5].

S2.7 Live/Dead Analysis of Core-Sheath Fibre Scaffolds

Samples were stained with 0.001% Syto-9 (Invitrogen) and 0.0015% propidium iodide (Invitrogen) at 37 °C for 30 min [6]. Samples were imaged using an upright Zeiss LSM 510 confocal microscope. Three fields of view were taken for each sample, and cells were counted using the ITCN cell counter software on ImageJ (threshold = 40 to 90). The average number of live cells versus dead cells ± standard deviation (SD) per sample were plotted on a graph as well as expressed as a percentage of cell viability [6].

S3 Results and Discussion

S3.1 Characterisation of Polyhydroxyalkanoates

S3.1.1 Molecular composition of Polyhydroxyalkanoates by Fourier-Transform Infrared (FT-IR)

The molecular structure of the polymers is validated by a Fourier-Transform Infrared (FT-IR) spectroscopy (Figure S1). The key functional groups are detected; stretching of terminal alkyl group (-CH_3) of the side chain on PHA-produced by *P. mendocina* CH50 is visible at around 2800 cm^{-1}, along with the asymmetrical stretching of -CH_2- chain at around 2,900 cm^{-1}. Meanwhile, for P(3HB) produced by *B. subtilis,* OK2 only exhibited the -CH_3 stretching at almost 3,000 cm^{-1} wavenumbers because the side chain contains only one carbon. As polyester, a typical carbonyl group signal can be observed around 1,700 cm^{-1} for both polymers, followed by distinguishable fingerprint regions between 1,500 cm^{-1} and 600 cm^{-1}, indicating the presence of an extended linear ester chain [7]. This analysis serves as an initial confirmatory characterisation of the polymers produced are indeed PHAs.

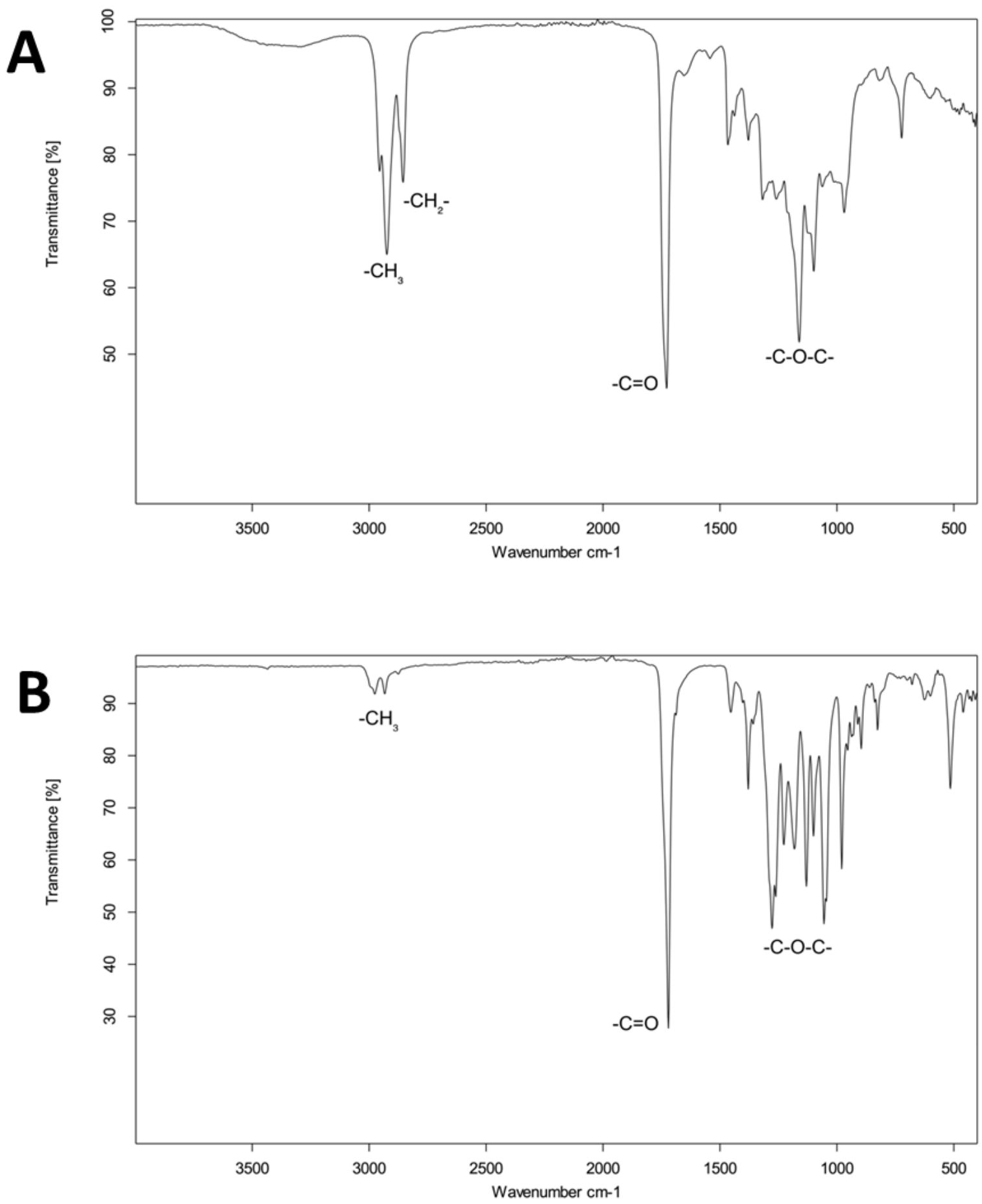

Figure S1: Fourier-Transform Infrared (FT-IT) spectra of (A) mcl-PHA from *P. mendocina* CH50, and (B) P(3HB) from *B. subtilis* OK2.

S3.1.2 Molecular composition of Polyhydroxyalkanoates by Nuclear Magnetic Resonance (NMR)

Nuclear Magnetic Resonance (NMR), has also been carried out to confirm the molecular composition of the PHAs further (Figure S2). Two different NMRs were selected: proton (^{1}H), which detects the implicit hydrogen molecules, and carbon (^{13}C), which detects the carbon molecules within the polymer chain.^{1}H NMR expresses the presence of two hydrogen atoms attached to the alpha carbon as a unique multiplet around 2.5 ppm, visible in both polymer spectra. The single hydrogen on the beta carbon, where the side chain branches, is also found at around 5.1 ppm. The mcl-PHA from *P. mendocina* exhibited three additional hydrogen atoms signals representing those on the terminal alkyl group (-CH_3), the -CH_2- bonded with the beta carbon the following -CH_2-, at around 0.8 ppm, 1.2 ppm and 1.5 ppm respectively (Figure S2A). Meanwhile, the P(3HB) from *B. subtilis* OK2 is expected to have only a terminal alkyl group of -CH_3, which corresponds with a signal representing the three hydrogen atoms at

around 1.2 ppm on the ^{1}H NMR spectrum (Figure S2B). ^{13}C NMR, on the other hand, verifies the conformation of carbon atoms within the polymers. Like the ^{1}H NMR, alpha and beta carbon signals are present at around 40 ppm and 70 ppm, respectively. In addition, carbonyl carbon (-C=O) is present at around 170 ppm, further de-shielded due to the high electronegative environment contributed by an oxygen atom. The mcl-PHA spectrum exposed signals of the side chain components, spanning from 10 ppm to 35 ppm, indicating the aliphatic chain branched from the beta carbon (Figure S2A). Meanwhile, P(3HB), as a simpler PHA, shows only the signal of the terminal alkyl of $-CH_3$ at around 20 ppm (Figure S2B).

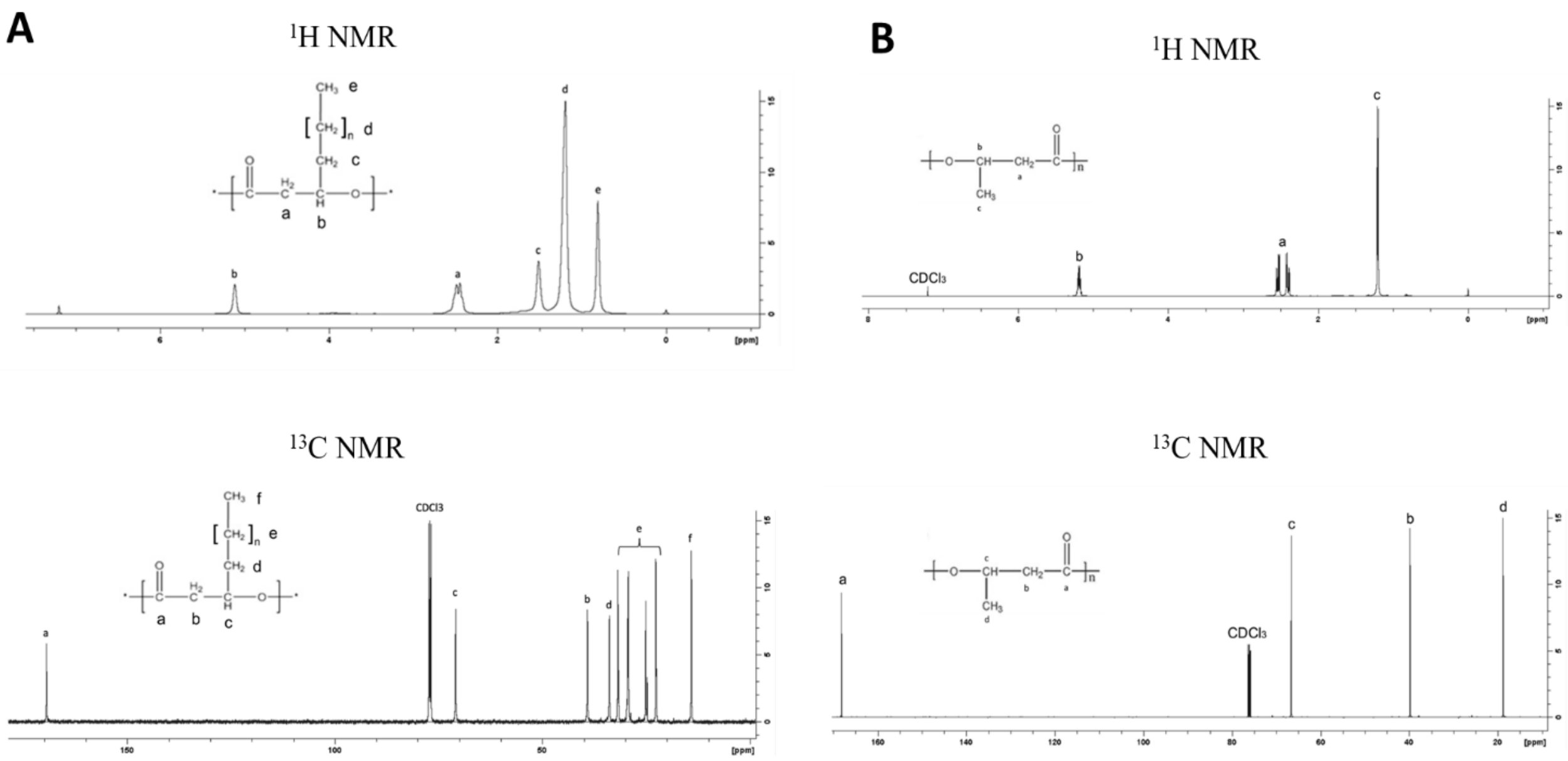

Figure S2: Proton and Carbon Nuclear Magnetic Resonance (NMR) spectra analysis of (A) mcl-PHA from *P. mendocina* CH50 and (B) P(3HB) from *B. subtilis* OK2.

S3.1.3 Thermal properties of Polyhydroxyalkanoates by Differential Scanning Calorimetry (DSC)

PHAs are known as thermoplastic materials, which melt when heated and harden when cooled down. Both polymers experienced glass transition (T_g) and endothermic melting (T_m) phase when subjected to Differential Scanning Calorimetry (DSC) analysis. Mcl-PHA had -45.1 °C for T_g and 4.9 °C for scl-PHA, a stark difference due to their molecular structure. This is also consistent for their T_m, with 52.8 °C for mcl-PHA and 165.1 °C for scl-PHA, significantly higher for having shorter chain that contributing to denser molecular packing and more crystalline, compared to the mcl-PHA which widely known to be type of amorphous polymer (Table S2) [8]. Corresponding to this, crystallisation exothermic process only happened in scl-PHA, at 66.0 °C further exhibit this characteristic (Table S2). These observations are coherent with established works in similar polymers [9].

Table S2: Thermal properties of the polymers produced by respective strains, obtained with Differential Scanning Calorimetry (DSC) analysis.

Strain	Polymer	Thermal Parameters, °C*		
		T_g	T_c**	T_m
P. mendocina CH50	Mcl-PHA	-45.1	-	52.8
B. subtilis OK2	P(3HB)	4.9	66.0	165.1

* T_g: Glass transition temperature; T_c: Crystallisation temperature; T_m: Melting temperature

** During cooling cycle.

S3.1.3 Molecular weight of Polyhydroxyalkanoates by Gel permeation chromatography (GPC)

The molecular weight of the polymer was studied using GPC. Both molecular weights of the polyhydroxyalkanoates produced were above 300,000 Da, exhibiting high molecular weights, with a wide molecular weight distribution (table S3). This was also seen in our previous study [1].

Table S3. Molecular weight of P(3HB) and MCL-PHA

	MCL-PHA	P(3HB)
Weight average Molecular weight (M_w)	348000 Da	455500 Da
Number average Molecular weight (M_n)	92000 Da	112745 Da

Polydispersity index (PDI)	4.4	4.1

S3.1 Fibre Production Rate Analysis

The ratio between the diameter of the core and the sheath layer of each strand of fibre was (7:3 at 1,7,13 µm fibre diameter), (4:1 at 3,9,11 µm fibre diameter) and (9:1 at 5,15 µm fibre diameter) (Figure S3). Twin- layered C-S fibres had a production rate of 225 g h^{-1} (C-S PHA:PLA) and 208 g h^{-1} (C-S PLA:PHA) whereas PHA blend fibres had a production rate of 180 g h^{-1} and 151 g h^{-1} for PLA fibres (Figure S4). The overall production yield of the C-S PG was 225 g h^{-1}, which largely surpasses coaxial electrospinning with a production yield of 0.054 g h^{-1} (Table S4).

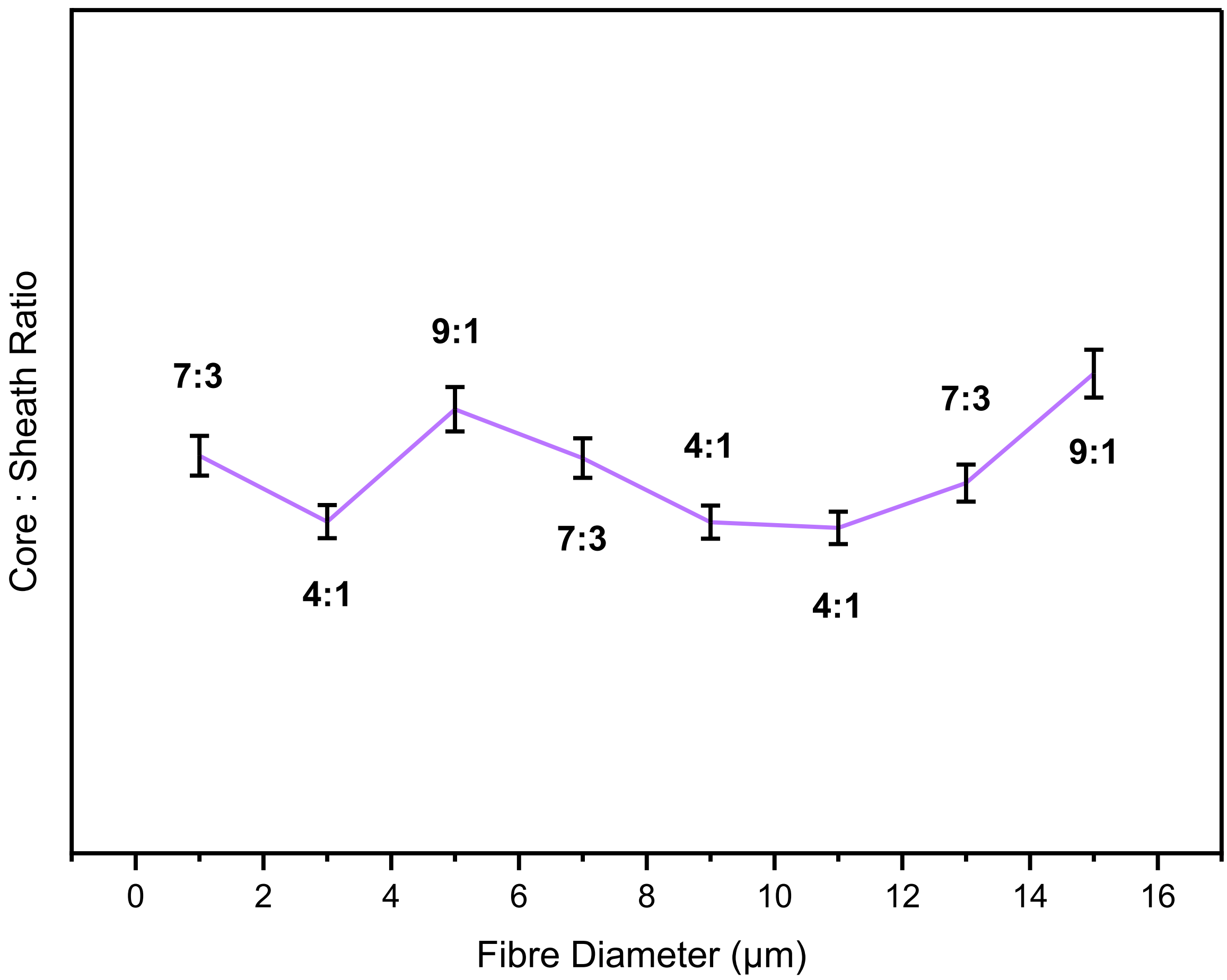

Figure S3. The ratio between the diameter of core and sheath of the fibres.

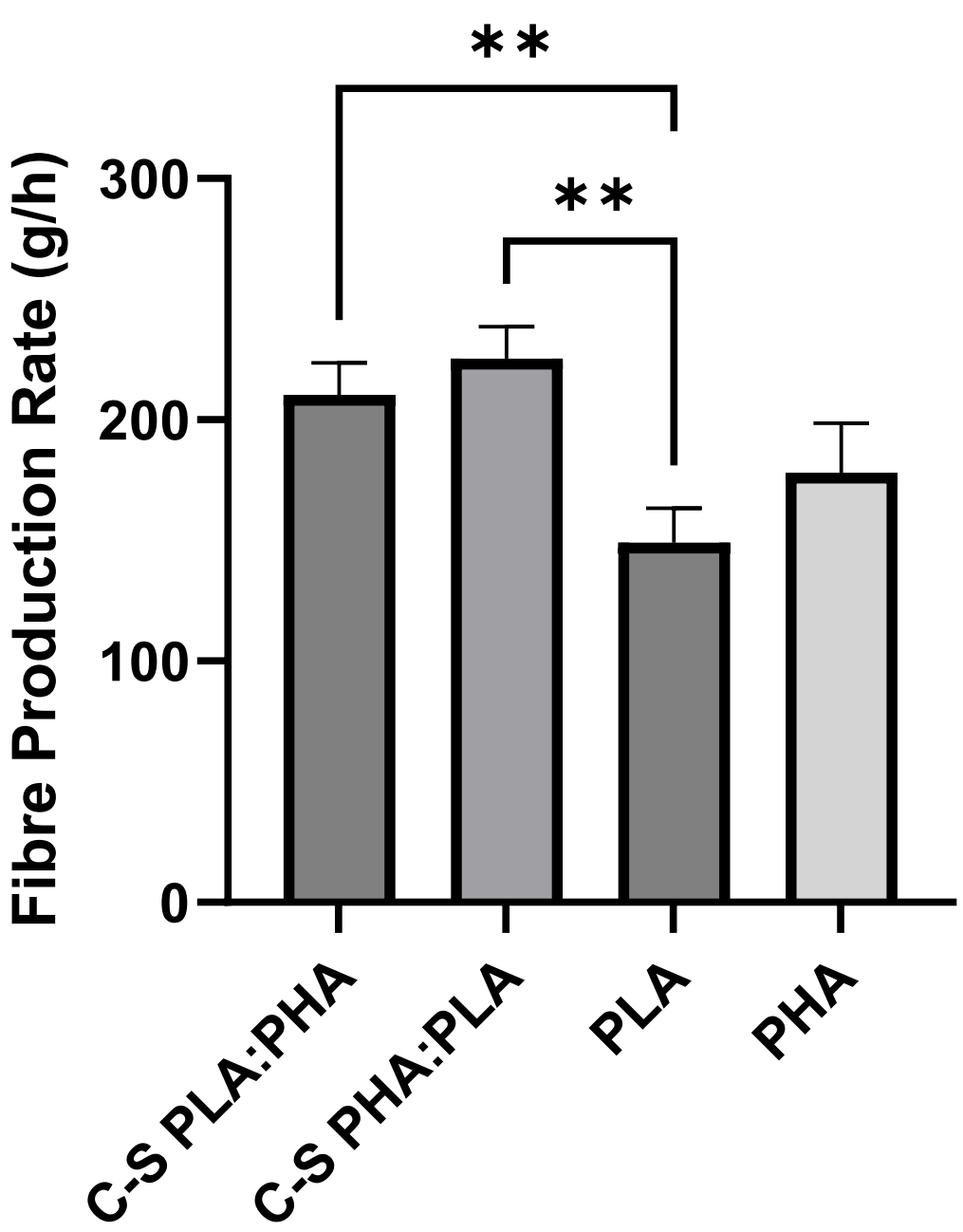

Figure S4. Mass production rate of C-S PG technique C-S PLA:PHA, C-S PHA:PLA, PLA and PHA fibres obtained at 0.1 MPa (mean ± SD, n=3 independent experiments; *p< 0.05 and **p<0.01).

Table S4. Comparison of manufacturing techniques for core-sheath twin layered fibre production yield.

**Spinning Technique**	**Production Rate [g h^{-1}]**
Coaxial Electrospinning	0.054 [10]
Core-Sheath Pressurised Gyration	225

S3.2 Ranges of Water Contact Angles

Figure S5 observed that the water contact angle of P(3HB) measured 78.5 ± 3.6° deeming it hydrophilic, whereas the mcl-PHA was hydrophobic, with a water contact angle of 102.5 ± 3.4°. When P(3HB) and mcl-PHA were blended together to form the PHA blend used in this work, the water contact angle measured at 83.4 ± 3.7° and the PLA was measured at 95.9 ± 5.2°. This deemed the PHA blend to be hydrophilic whereas the PLA to be relatively hydrophobic. The addition of P(3HB) to the mcl-PHA, formed a hydrophilic PHA blend (Figure S3), as seen in previous work by Lizarraga *et al.*[11] and Basnett *et al.* [5] using blends of P(3HB) with mcl-PHAs. Due to long aliphatic chains that are present in their chemical

structure, mcl-PHAs are hydrophobic in nature, and blending with a hydrophilic material decreases water contact angle and hence renders them to be more hydrophilic [12].

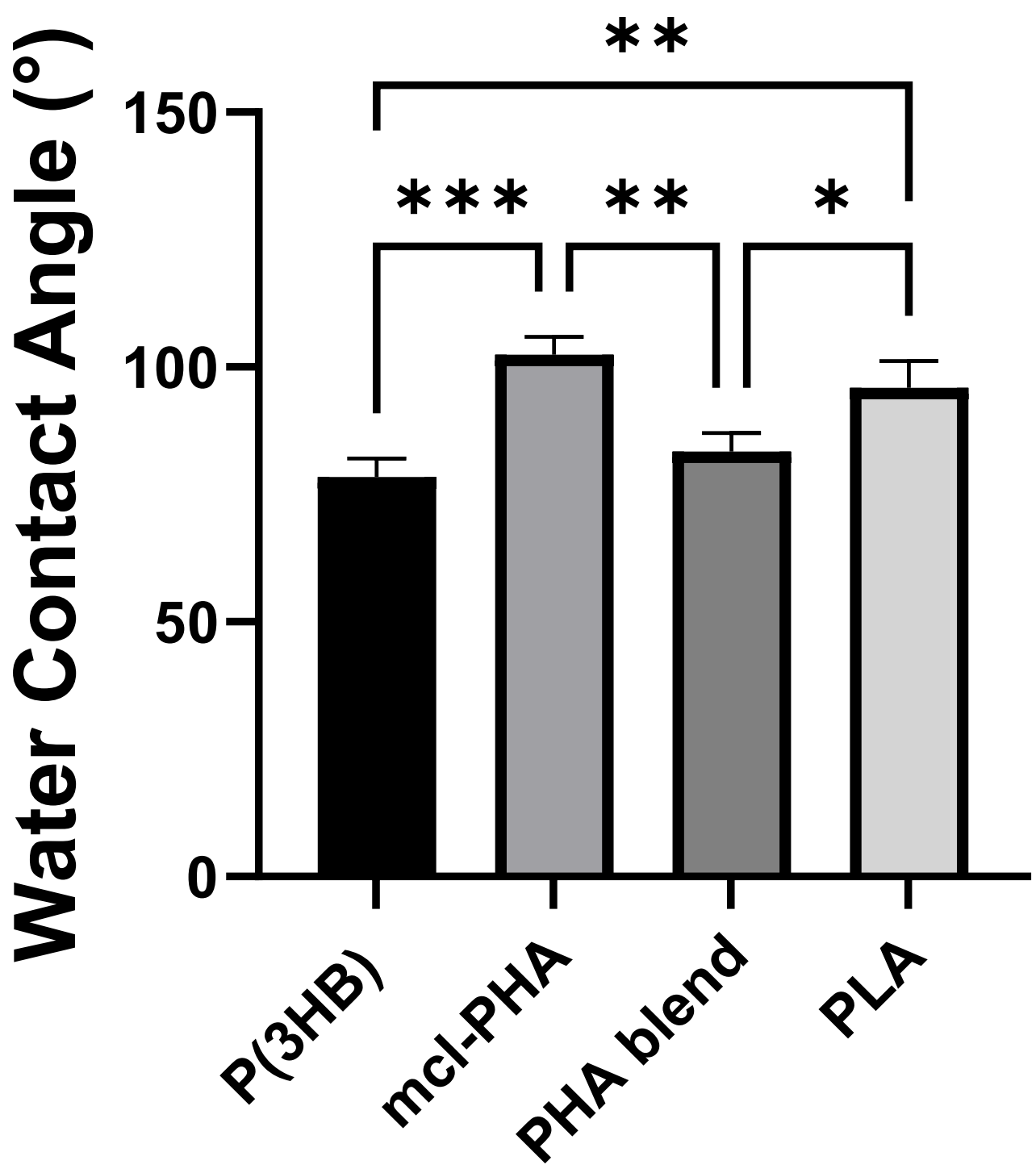

Figure S5. Water contact angle of P(3HB), mcl-PHA, PHA blend (P3HB/mcl-PHA 80/20) and PLA films. Results are shown as presented mean (mean ± SD, n=3 independent experiments; *p<0.05, **p<0.01 and ***p<0.001). A total of 9 samples, per condition, were analysed and a total of 27 contact angles measured per condition.

S3.3 Protein Adsorption Quantification on Core-Sheath Fibres

Total protein adsorbed onto the fibres was determined using the Bicinchoninic acid assay (Figure S6). The twin layered C-S PLA:PHA fibres exhibited significantly higher amounts of attached protein, 220.1 ± 25.5 μg/cm^2, compared to the C-S PHA:PLA fibres, 135.1 ± 22.4 μg/cm^2. PHA blend fibres promoted significant amounts of attached protein, 234.4 ± 20.1 μg/cm^2, compared to the PLA fibres, 120.3 ± 21.3 μg/cm^2.

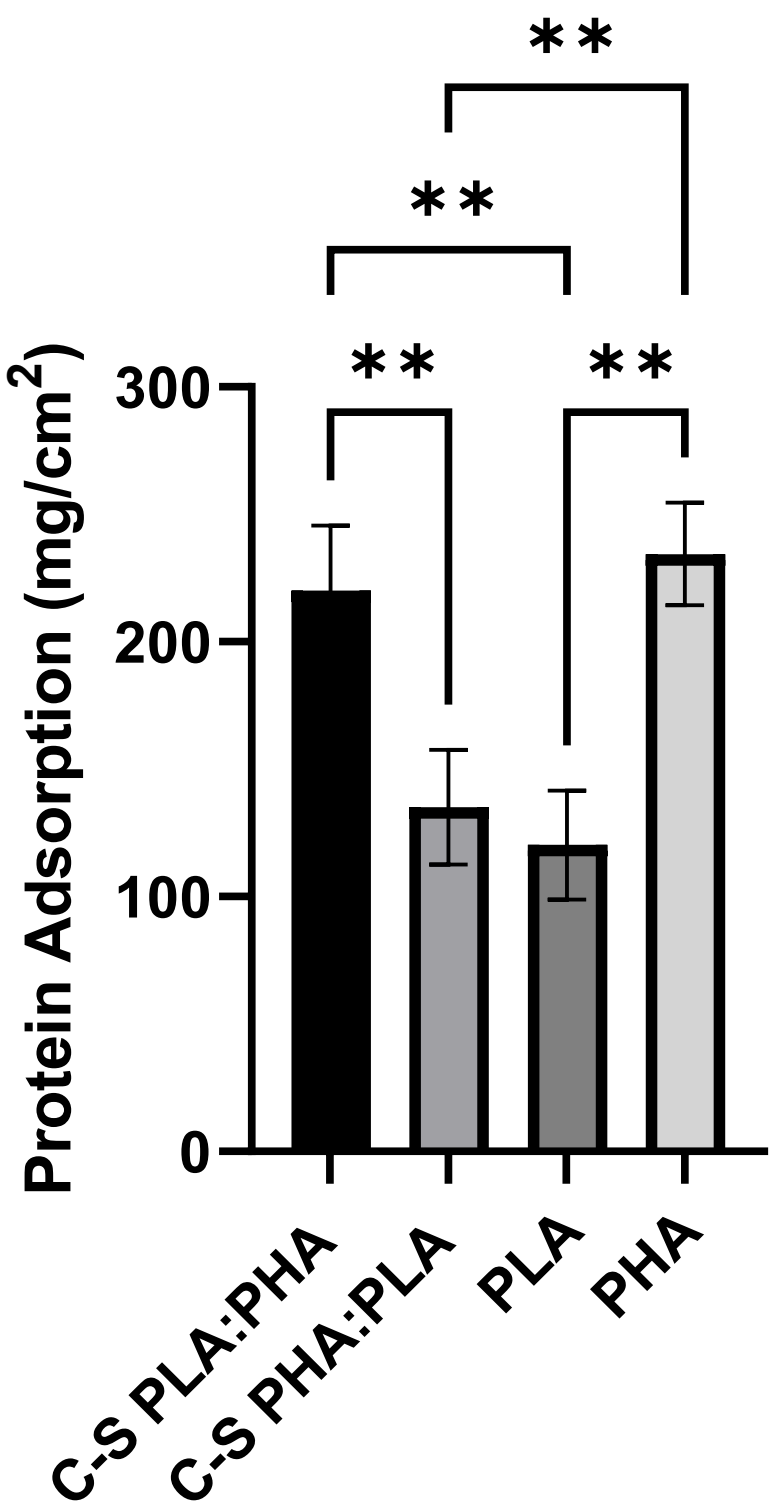

Figure S6. Protein adsorption quantified on C-S PLA:PHA, C-S PHA:PLA, PLA and PHA fibres after a 24-hour incubation (Data presented as mean ± SD, n=3 independent experiments, three replicates per n = 1 and three values taken per sample; **p<0.01).

S3.4 NG108-15 Neuronal Cell Viability

Figure S7A-E demonstrates live attached cells on all the fibres and TCP control, in which few dead cells can be observed. Fibres with a PHA sheath demonstrated significantly higher numbers of cells attached than with a PLA sheath, 149 ± 11.3 cells and 74 ± 41.9 cells, respectively (Figure S7F). Both PHA blend fibres, and C-S PLA:PHA fibres promoted significantly higher numbers of live NG108-15 cell attachment, 149 ± 11.3 and 137 ± 24.1 cells, compared to the PLA fibres, 81 ± 27.4 cells. All substrates demonstrated cell viability above 95% deeming them all biocompatible (Figure S7G). No significant differences were detected between data sets.

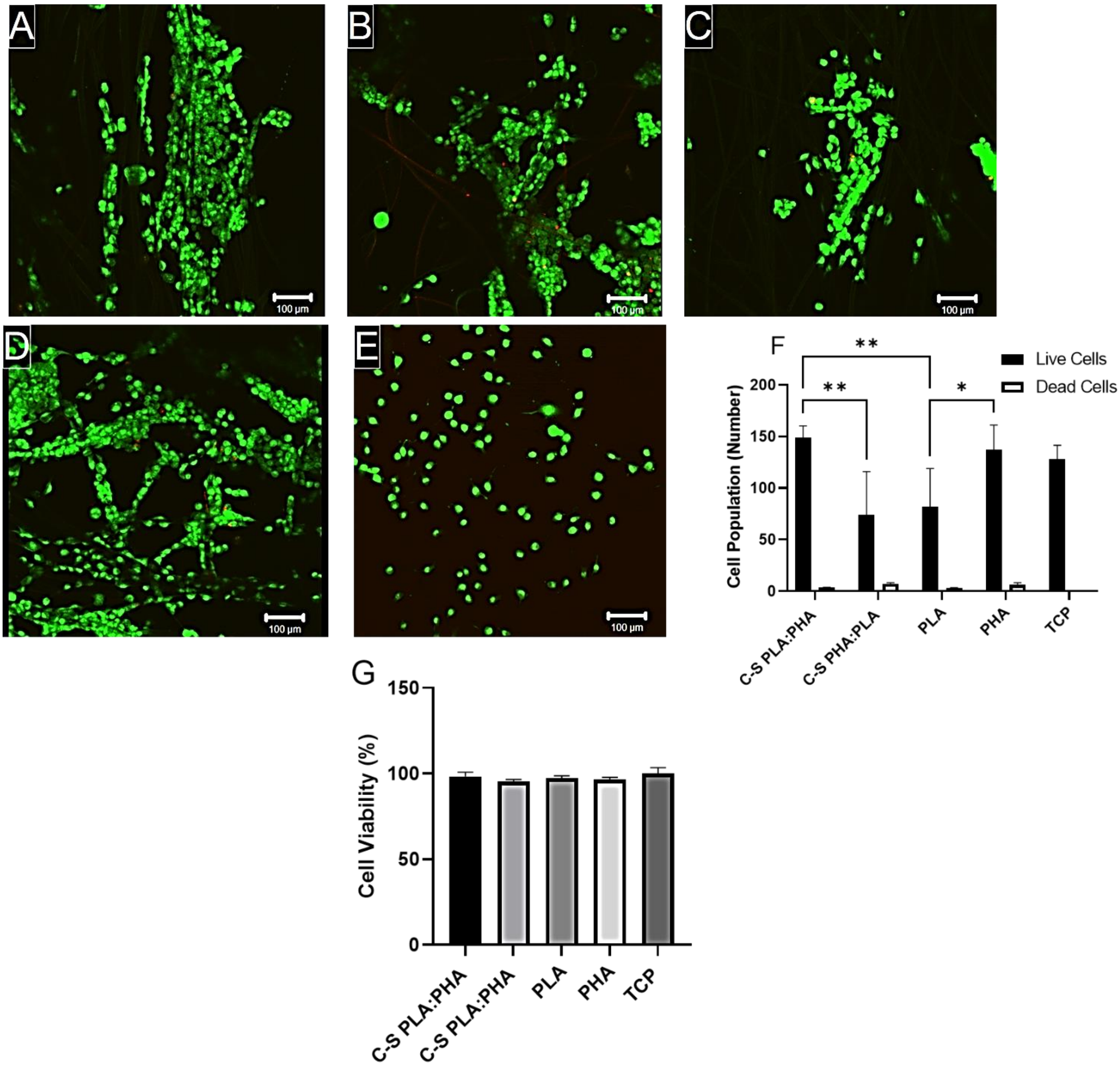

Figure S7. Confocal micrographs illustrating NG108-15 neuronal cell viability cultured on: (A) C-S PLA:PHA, (B) C-S PHA:PLA, (C) PLA and (D) PHA fibres. (E) Tissue culture plastic control (Scale bar = 100 μm). Cells labelled with syto 9 (green= live cells) and propidium iodide (red= dead cells). F) Number of live cells versus dead cells per sample and G) live cell analysis expressed as a percentage (mean ± SD, n=3 independent experiments; *p< 0.05 and **p<0.01).

S3.5 C2C12 Myoblast Cell Viability

Figure S8A-E demonstrates live attached cells on all the fibres and TCP control, in which few dead cells can be observed. C-S PLA: PHA fibres supported significantly higher numbers of live cell attachment, 273 ± 54.4 cells, compared to the PHA blend fibres, 67 ± 49.9 cells (Figure S8F), and was not significantly different to the TCP control, indicating excellent cell growth on the C-S PLA: PHA fibres. Significantly higher number of live cells were attached to the TCP control, 393 ± 202.9 cells, compared to the twin-layered C-S PHA:PLA, 189 ± 72.8 cells, PLA fibres, 95 ± 84.1 cells and the PHA blend fibres. All substrates demonstrated cell viabilities of above 95% deeming them all biocompatible (Figure S8G). No significant differences were detected between data sets.

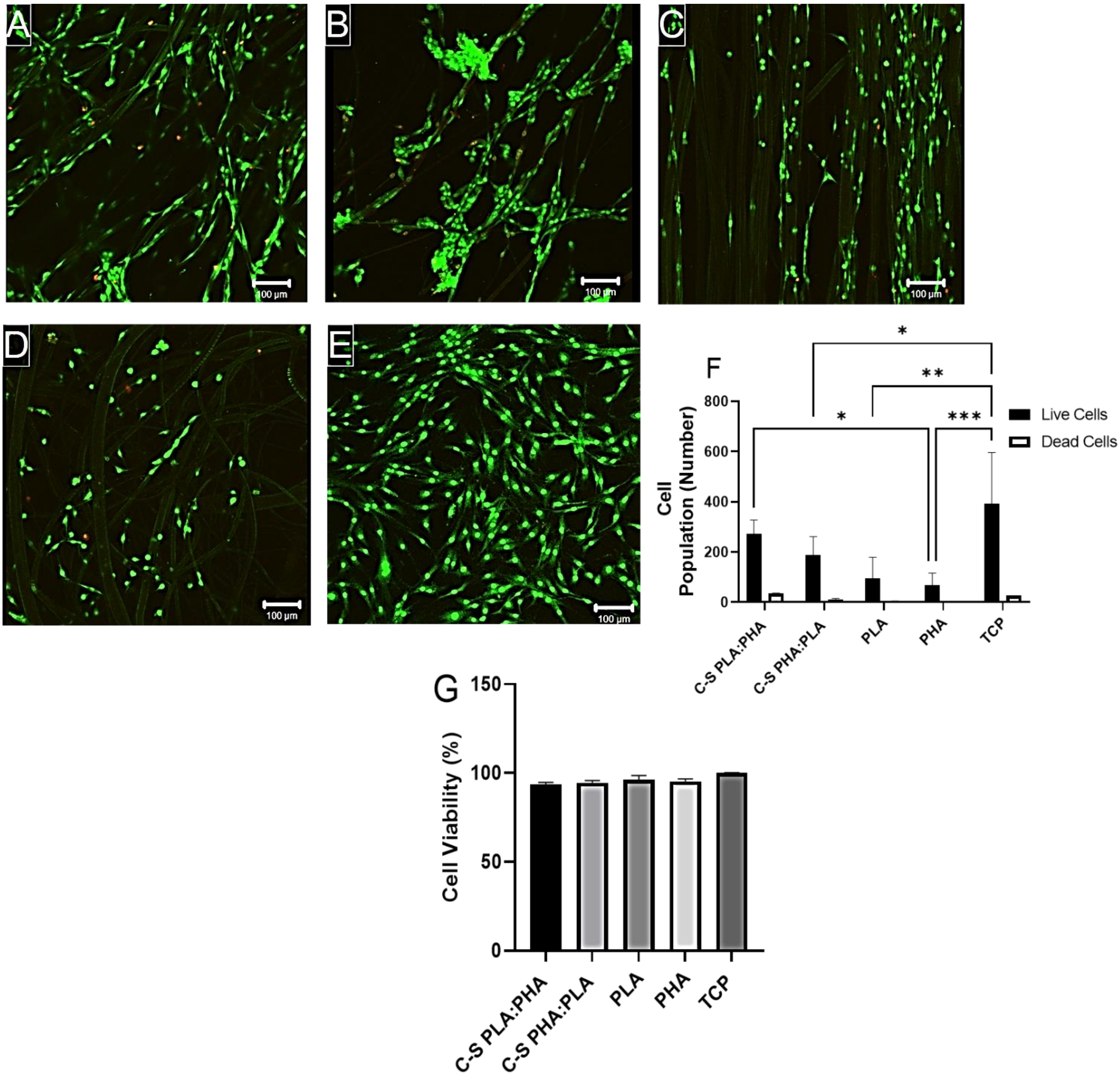

Figure S8. Confocal micrographs illustrating C2C12 myoblast cell viability cultured on: (A) C-S PLA:PHA, (B) C-S PHA:PLA, (C) PLA and (D) PHA fibres. (E) Tissue culture plastic control (Scale bar = 100 μm). Cells labelled with syto 9 (green= live cells) and propidium iodide (red= dead cells). F) Number of live cells versus dead cells per sample and G) live cell analysis expressed as a percentage (mean ± SD, n=3 independent experiments; *$p < 0.05$, **$p<0.01$ and ***$p<0.001$).

S3.6 Human Primary Osteoblast Viability

Figure S9F demonstrates that PLA fibres and twin layered C-S PLA: PHA fibres, significantly promoted higher numbers of osteoblast attachment, 261 ± 26.8 cells and 252 ± 77.1 cells, compared to the twin layered C-S PHA: PLA fibres, 154 ± 44.5 cells. All substrates

demonstrated cell viabilities of above 95% deeming them all biocompatible (Figure S9G). No significant differences were detected between data sets.

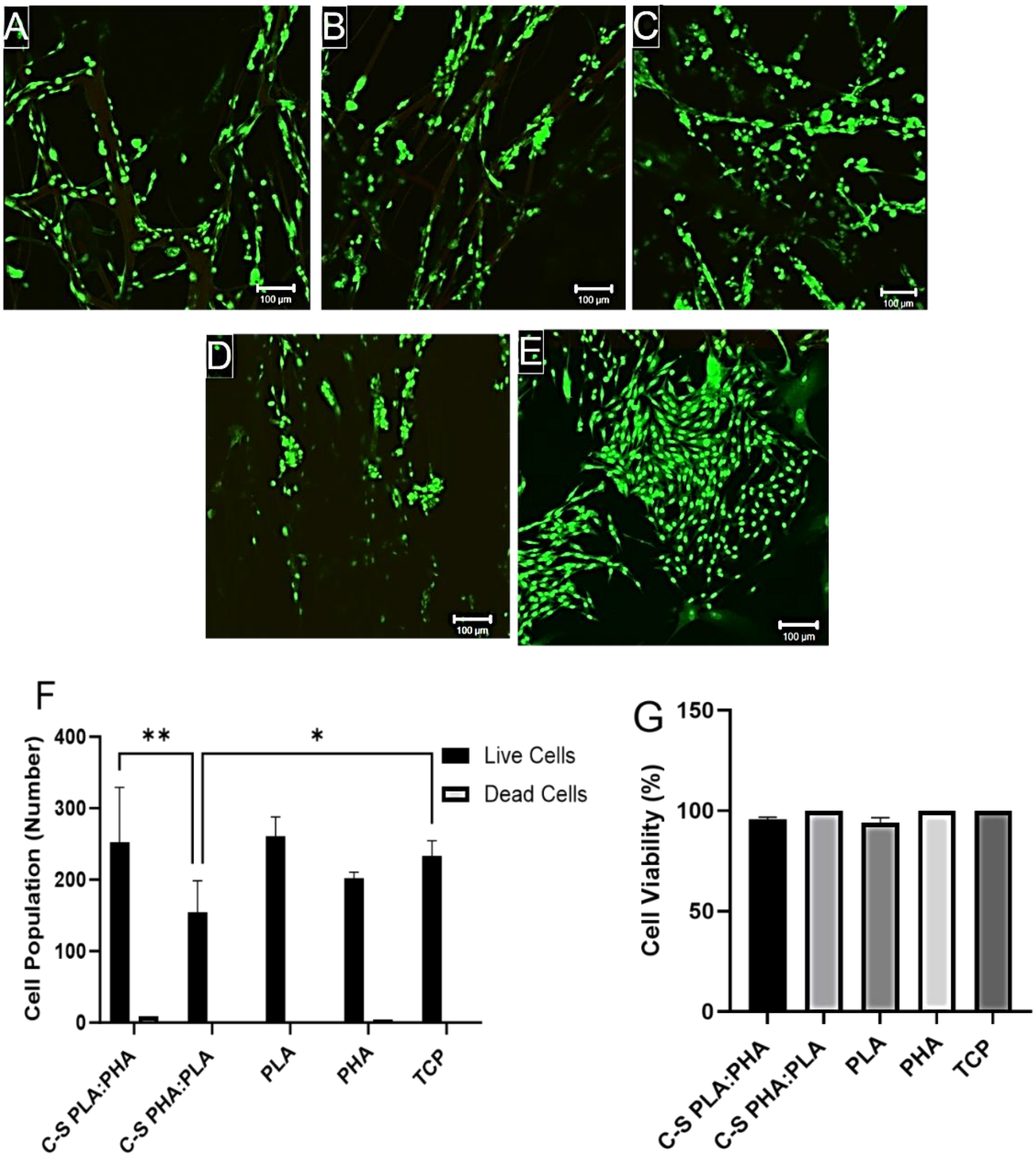

Figure S9. Confocal micrographs illustrating Human primary osteoblast cell viability cultured on: (A) C-S PLA:PHA, (B) C-S PHA:PLA, (C) PLA and (D) PHA fibres. (E) Tissue culture plastic control (Scale bar = 100 µm). Cells labelled with syto 9 (green= live cells) and propidium iodide (red= dead cells). F) Number of live cells versus dead cells per sample and G) live cell analysis expressed as a percentage (mean ± SD, n=3 independent experiments; *$p< 0.05$ and **$p<0.01$).

S3.7 BRIN-BD11 Rat Pancreatic Cell Viability

Figure S10A-E demonstrated that significantly more attached live cells were observed on the twin layered C-S PLA:PHA fibres, 290 ± 17.8 cells, and the tissue culture plastic control, 481

± 30.3 cells, compared to cells counted on the twin layered C-S PHA:PLA fibres, 188 ± 31.8 cells, PLA fibres, 181 ± 22.3 cells, and the PHA blend fibres, 220 ± 66.1 cells (Figure S10F). All substrates demonstrated cell viabilities of above 95% deeming them all biocompatible (Figure S10G). No significant differences were detected between data sets.

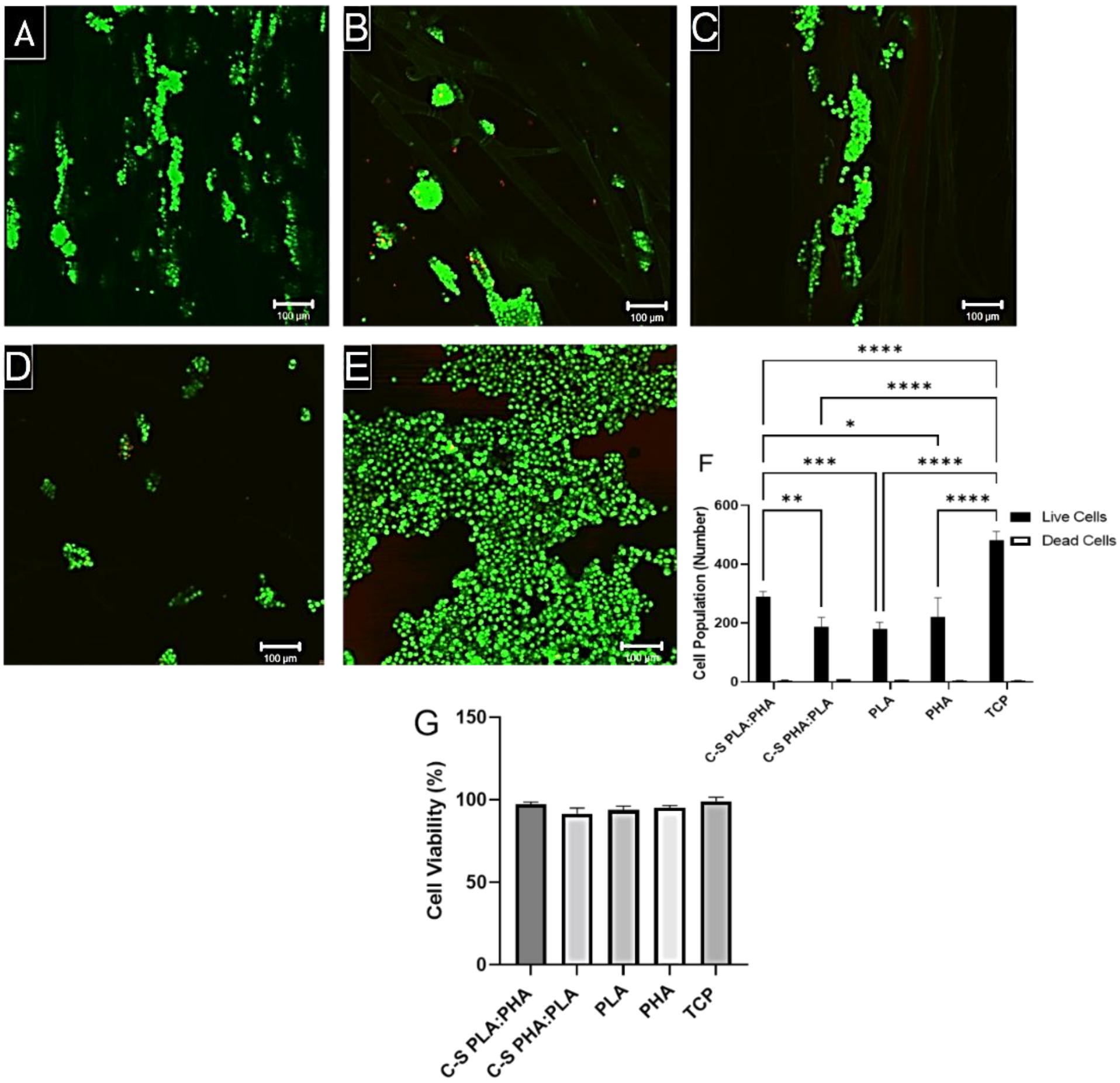

Figure S10. Confocal micrographs illustrating BRIN-DB11 rat pancreatic cell viability cultured on: (A) C-S PLA:PHA, (B) C-S PHA:PLA, (C) PLA and (D) PHA fibres. (E) Tissue culture plastic control (Scale bar = 100 µm). Cells labelled with syto 9 (green= live cells) and propidium iodide (red= dead cells). F) Number of live cells versus dead cells per sample and G) live cell analysis expressed as a percentage (mean ± SD, n=3 independent experiments; *p< 0.05, **p<0.01, ***p<0.001 and ****p<0.0001).

S3.8 Collagen IV Formation from Conditionally Immortalised Human Glomerular Podocytes (CIHP) Viability

The CIHPs adhered to all fibre substrates in which few dead cells were observed (Figure S11A-E). Significantly more attached live cells were observed on the PHA blend fibres, 264 ± 37.5 cells, compared to the mono PLA fibres, 56 ± 14.5 cells, twin layered C-S PLA:PHA fibres, 57 ± 14.5 cells, and the twin layered C-S PHA:PLA fibres, 66 ± 17.3 cells (Figure

S11F). The highest number of live cells were observed on the TCP control, 688 ± 45.5 cells, which also had the highest number of dead cells, 100 ± 17.5. Due to increased numbers of dead cells, TCP had a cell viability of 87.1 ± 2.8 %, as observed in Figure S11G. The C-S PLA: PHA fibres and the PLA blend fibres had cell viabilities of over 90%, 91.8 ± 5.7 %, and 90.7 ± 8.6 %, respectively, deeming them highly biocompatible

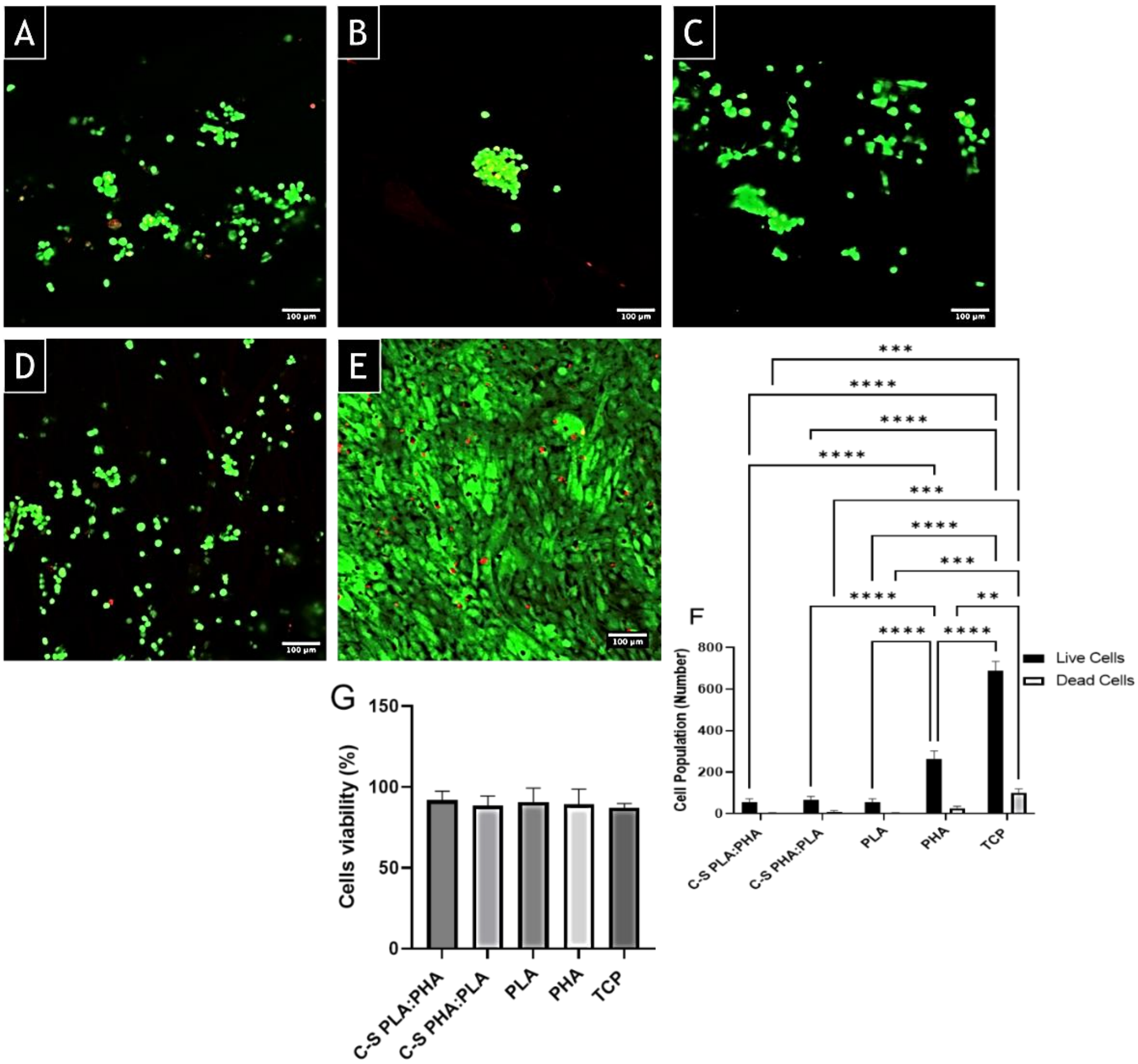

Figure S11. Confocal micrographs illustrating conditionally immortalised human glomerular podocytes viability cultured on: (A) C-S PLA:PHA, (B) C-S PHA:PLA, (C) PLA and (D) PHA fibres. (E) Tissue culture plastic control (Scale bar = 100 µm). Cells labelled with syto 9 (green= live cells) and propidium iodide (red= dead cells). F) Number of live cells versus dead cells per sample and G) live cell analysis expressed as a percentage (mean ± SD, n=3 independent experiments; **p<0.01, ***p<0.001 and ****p<0.0001).

S3.9 Collagen IV Formation from Conditionally Immortalised Glomerular Endothelial cells (CiGEnCs) and Viability

All fibre substrates supported collagen type IV formation from Conditionally Immortalised Glomerular Endothelial cells (CiGEnCs) (Figure S12A-E). No significant differences of collagen type IV expression were detected between any of the fibre substrates and TCP control. Collagen type IV mean fluorescent intensity (%) on the twin layered C-S PLA:PHA fibres, twin layered C-S PHA:PLA fibres, PLA fibres, PHA blend fibres and tissue culture plastic control was 21.3 ± 2.4%, 16.4 ± 4.8%, 16.3 ± 6.8%, 26.5 ± 6.1% and 22.6 ± 6.1%, respectively (Figure S12F).

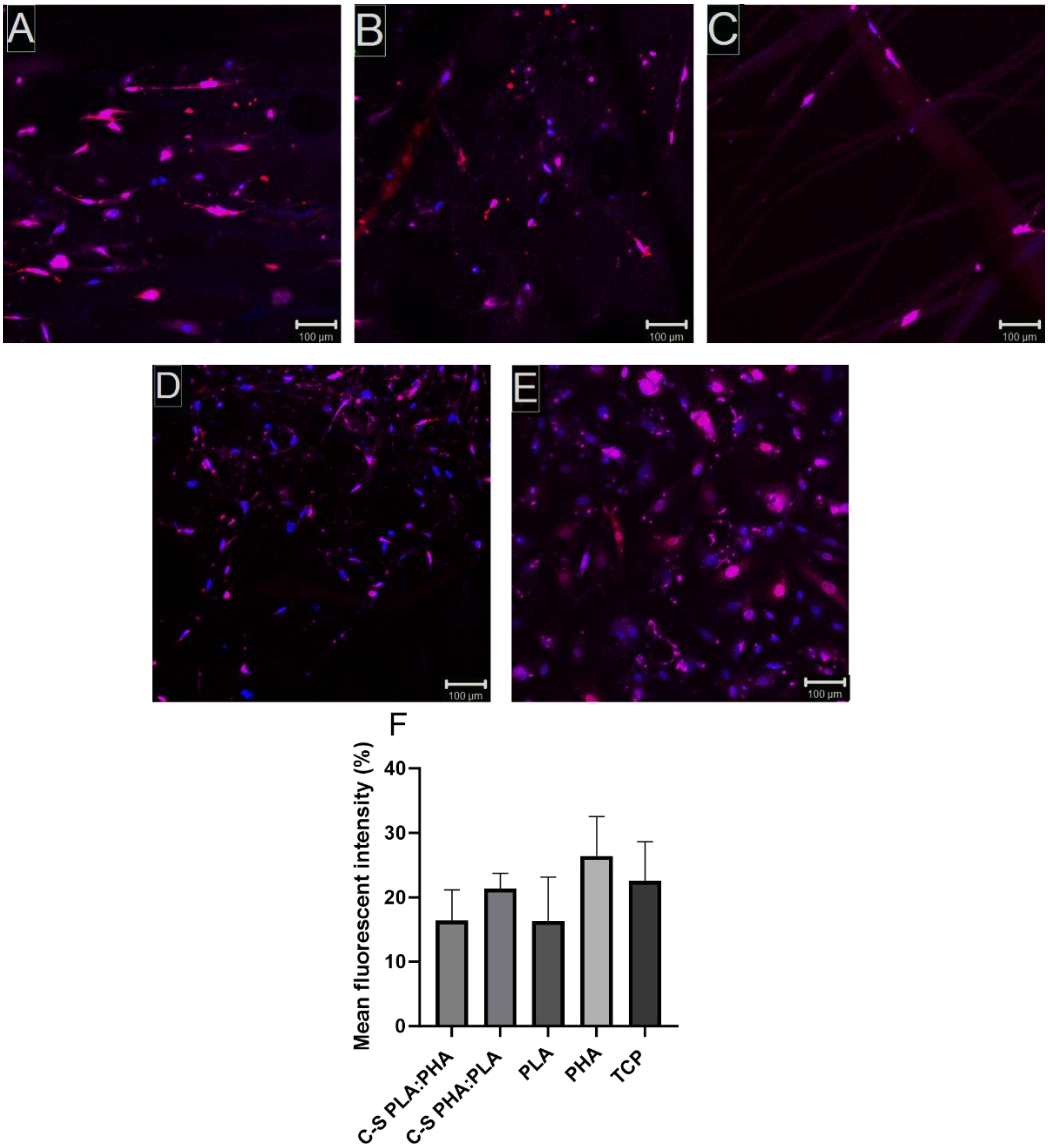

Figure S12. Confocal micrographs illustrating conditionally immortalised glomerular endothelial cells stained for DAPI (blue), mcCherry (red) and collagen IV (purple) cultured on: (A) C-S PLA:PHA, (B) C-S PHA:PLA, (C) PLA and (D) PHA fibres. (E) Tissue culture plastic control (Scale bar = 100 µm). Cells labelled with syto 9 (green= live cells) and propidium iodide (red= dead cells). F) Mean fluorescent intensity of collagen IV (mean ± SD, n=3 independent experiments).

CiGEnCs attached to all substrates in which few dead cells were observed (S13A-E). Significantly more attached live cells were observed on the twin layered C-S PHA:PLA fibres, 46 ± 4.3 cells, and the mono PLA fibres, 38 ± 2.3 cells, compared to the twin layered C-S PLA:PHA fibres, 19 ± 1.2 cells (Figure S13F). The highest number of live cells on the fibre substrates were observed on the PHA blend fibres, 76 ± 5.2 cells. All substrates demonstrated cell viabilities of above 90% deeming them all biocompatible (Figure S13G). No significant differences were detected between data sets.

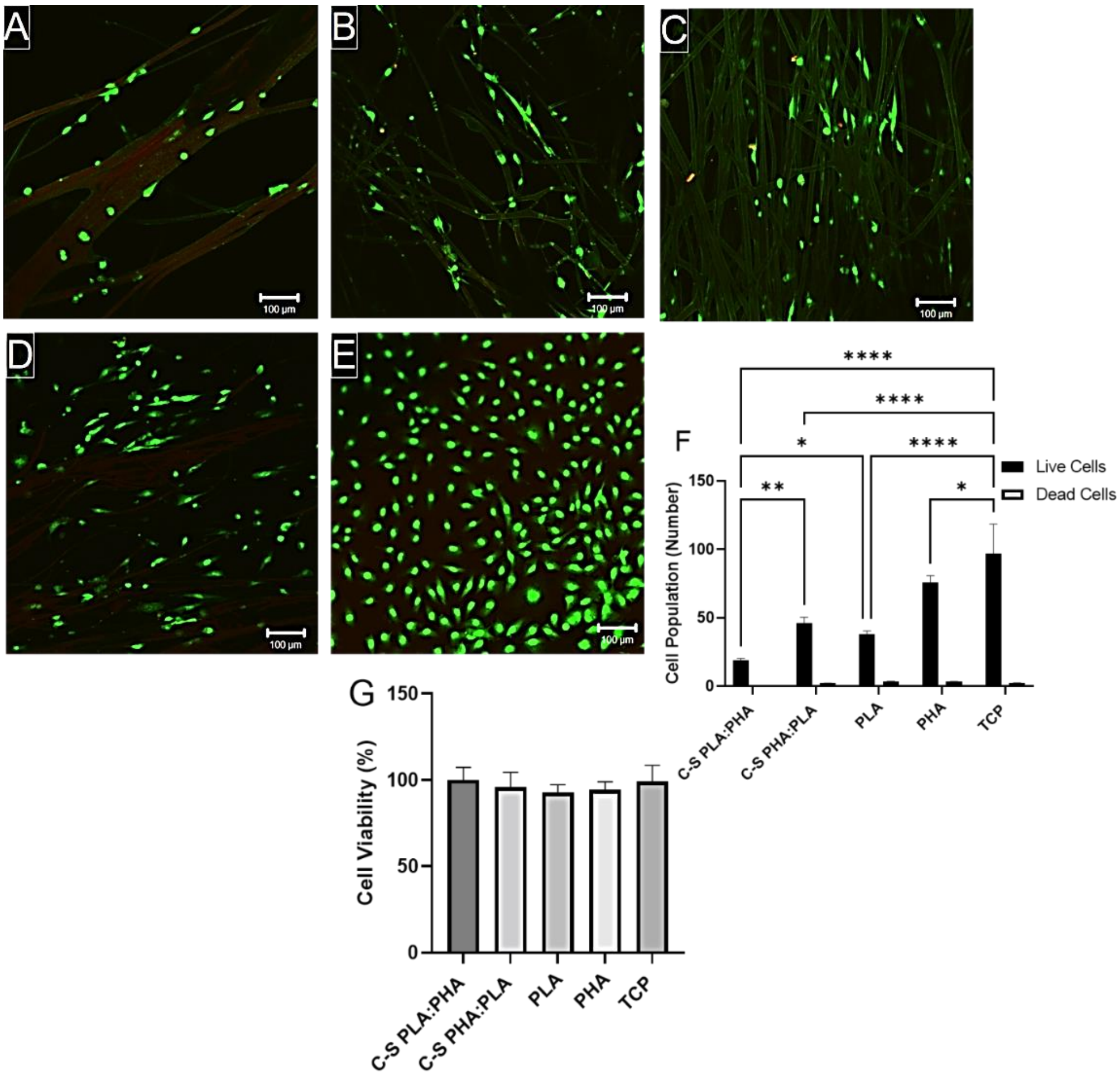

Figure S13. Confocal micrographs illustrating conditionally immortalised glomerular endothelial cell viability cultured on: (A) C-S PLA:PHA, (B) C-S PHA:PLA, (C) PLA and (D) PHA fibres. (E) Tissue culture plastic control (Scale bar = 100 μm). Cells labelled with syto 9 (green= live cells) and propidium iodide (red= dead cells). F) Number of live cells versus dead cells per sample and G) live cell analysis expressed as a percentage (mean ± SD, n=3 independent experiments; *p< 0.05, **p<0.01 and ****p<0.0001).

S3.10 Immortalised Human Normal Keratinocyte Cell (HaCaTs) Phenotype and Viability

Figure S14A-E demonstrates confocal micrographs of HaCaTs cultured on all fibre substrates with cells positively stained for keratin 10.

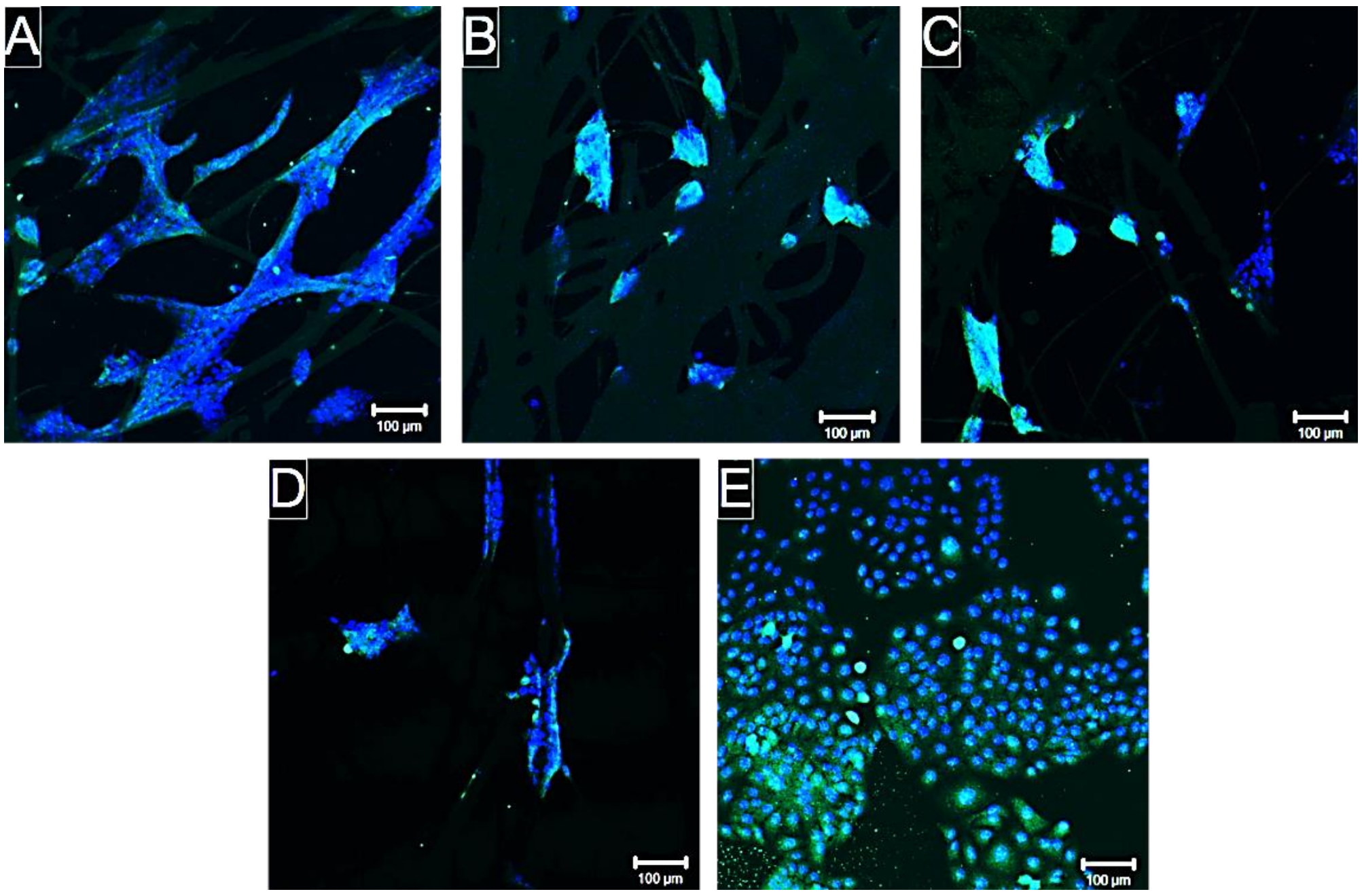

Figure S14. Confocal micrographs illustrating Immortalised Human normal keratinocyte cells stained for stained for cytokeratin (green) and DAPI (blue) on: (A) C-S PLA:PHA, (B) C-S PHA:PLA, (C) PLA and (D) PHA fibres. (E) Tissue culture plastic control (Scale bar = 100 µm).

All substrates supported live viable HaCaT cell attachment (Figure S15A-E). Significantly more attached live cells were observed on the twin layered C-S PLA: PHA fibres, 114 ± 43.5 cells, and the tissue culture plastic control, 135 ± 32.3 cells, compared to the C-S PHA:PLA fibres, 46 ± 21.3 cells (Figure S15F). No significant differences were detected between C-S PLA: PHA fibres and the tissue culture plastic control compared to the PLA fibres and PHA blend fibres. All substrates demonstrated cell viabilities of above 90% deeming them all biocompatible (Figure S15G). No significant differences were detected between data sets. C-S fibres also showed potential for use in skin tissue engineering applications [13]. Immortalised Human keratinocyte cells (HaCaTs) cultured on all fibre scaffolds stained positively for Keratin 10, and the twin layered C-S PLA: PHA fibres significantly supported increased live viable HaCaTs compared to the other fibre conditions. Previous work has shown that PHA blends, of P(3HB) with mcl-PHAs, support keratinocyte phenotype, proliferation and growth. HaCaTs stained positive for cytokeratin after 7 days of being cultured on P(3HB)/P(3HO-*co*-3HD) nanofibers in the study by Kalaoglu-Altan *et al.*[14] and Azimi *et al*. demonstrated

P(3HB)/P(3HO-*co*-3HD) blend nanofibers supported HaCaT proliferation, more so than with the addition of chitin into the fibres [15]. Though PLA has also been reported to support HaCaT culture, modifying PLA with various surface treatments, such as plasma polymerisation, has been shown to significantly improve cell phenotype, viability and proliferation as compared to neat PLA. Bacakova *et al.* [13] noted an increase in Kertain 5 expression from HaCaTs on oxygen plasma treated PLA fibres, compared to untreated fibres. Bogdanova *et al.*[16] demonstrated increased HaCaT proliferation and viability, when incorporating gelatin into PLA fibres, decreasing surface hydrophobicity. These findings support our work that the addition of the PHA blend as a sheath to the PLA is beneficial for skin tissue engineering applications.

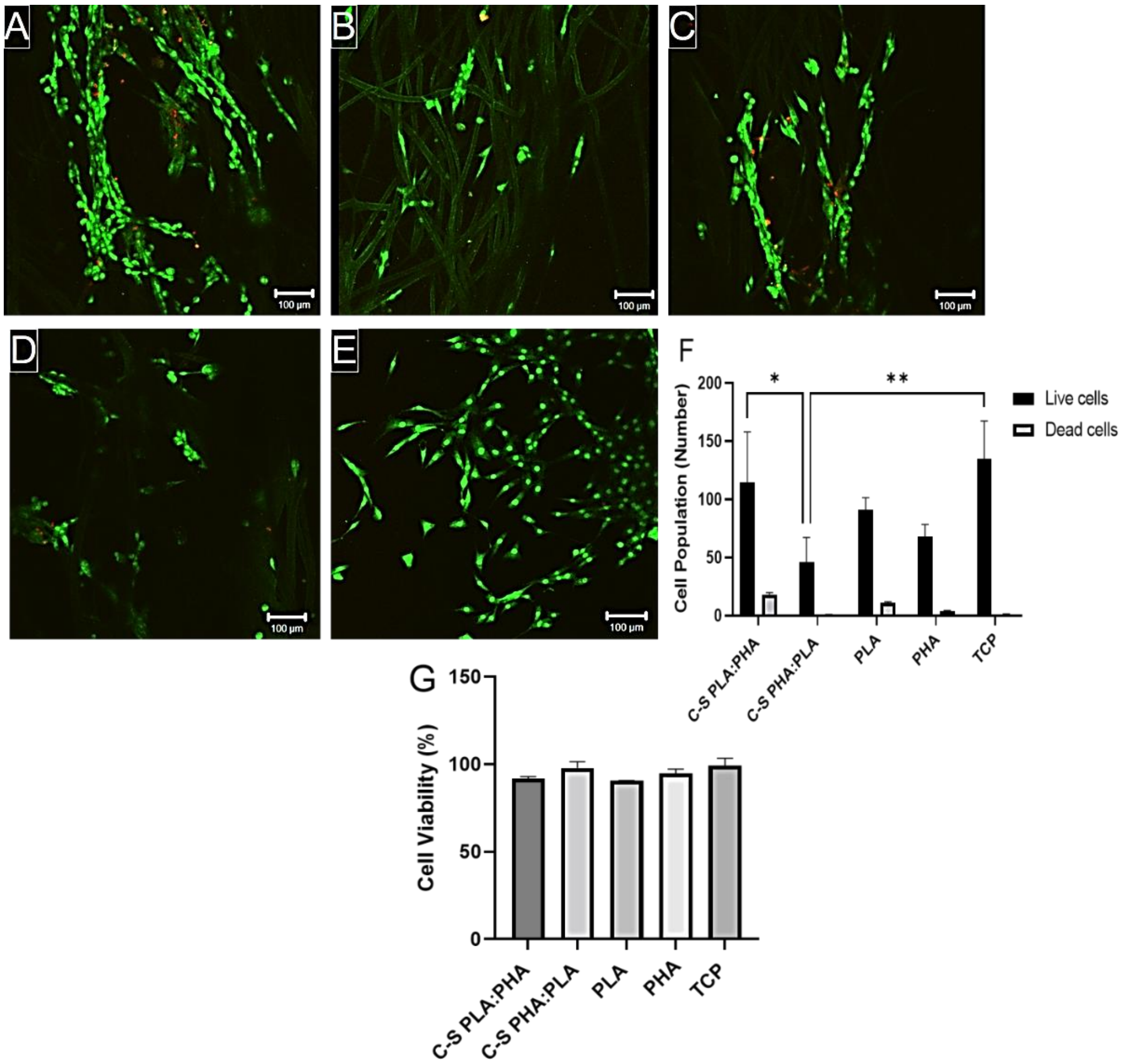

Figure S15. Confocal micrographs illustrating Immortalised Human normal keratinocyte cell viability cultured on: (A) C-S PLA:PHA, (B) C-S PHA:PLA, (C) PLA and (D) PHA fibres. (E) Tissue culture plastic control (Scale bar = 100 µm). Cells labelled with syto 9 (green= live cells) and propidium iodide (red= dead cells). F) Number of live cells versus dead cells per sample and G) live cell analysis expressed as a percentage (mean ± SD, n=3 independent experiments; *$p < 0.05$ and **$p < 0.01$).

References

1. P. Basnett, R. K. Matharu, C. S. Taylor, U. Illangakoon, J. I. Dawson, J. M. Kanczler, M. Behbehani, E. Humphrey, Q. Majid, B. Lukasiewicz, R. Nigmatullin, P. Heseltine, R. O. C. Oreffo, J. W. Haycock, C. Terracciano, S. E. Harding, M. Edirisinghe and I. Roy, *ACS Applied Materials & Interfaces*, 2021.
2. D. A. Gregory, A. T. R. Fricker, P. Mitrev, M. Ray, E. Asare, D. Sim, S. Larpnimitchai, Z. Zhang, J. Ma, S. S. V. Tetali and I. Roy, *Journal of Functional Biomaterials*, 2023, **14**, 40.
3. D. Singh, A. J. Harding, E. Albadawi, F. M. Boissonade, J. W. Haycock and F. Claeyssens, *Acta Biomaterialia*, 2018, **78**, 48-63.
4. H. Majd, A. Harker, M. Edirisinghe and M. Parhizkar, *Journal of Drug Delivery Science and Technology*, 2022, **72**, 103359.
5. P. Basnett, K. Y. Ching, M. Stolz, J. C. Knowles, A. R. Boccaccini, C. Smith, I. C. Locke, T. Keshavarz and I. Roy, *Reactive and Functional Polymers*, 2013, **73**, 1340-1348.
6. M. F. Daud, K. C. Pawar, F. Claeyssens, A. J. Ryan and J. W. Haycock, *Biomaterials*, 2012, **33**, 5901-5913.
7. P. Basnett, B. Lukasiewicz, E. Marcello, H. K. Gura, J. C. Knowles and I. Roy, *Microb Biotechnol*, 2017.
8. P. Wecker, X. Moppert, C. Simon-Colin, B. Costa and V. Berteaux-Lecellier.
9. R. Ortiz, P. Basnett, I. Roy and I. Quintana, *Polymers*, 2020, **12**.
10. H. Alenezi, M. E. Cam and M. Edirisinghe, *Applied Physics Reviews*, 2021, **8**, 041412.
11. L. R. Lizarraga-Valderrama, R. Nigmatullin, C. Taylor, J. W. Haycock, F. Claeyssens, J. C. Knowles and I. Roy, *Engineering in Life Sciences*, 2015, **15**, 612-621.
12. B. Lukasiewicz, P. Basnett, R. Nigmatullin, R. Matharu, J. C. Knowles and I. Roy, *Acta Biomaterialia*, 2018, **71**, 225-234.
13. M. Bacakova, F. Lopot, D. Hadraba, M. Varga, M. Zaloudkova, D. Stranska, T. Suchy and L. Bacakova, *Journal of Biomaterials Applications*, 2015, **29**, 837-853.
14. O. I. Kalaoglu-Altan, H. Baskan, T. Meireman, P. Basnett, B. Azimi, A. Fusco, N. Funel, G. Donnarumma, A. Lazzeri, I. Roy, S. Danti and K. De Clerck, *Materials*, 2021, **14**, 4907.
15. B. Azimi, L. Thomas, A. Fusco, O. I. Kalaoglu-Altan, P. Basnett, P. Cinelli, K. De Clerck, I. Roy, G. Donnarumma, M.-B. Coltelli, S. Danti and A. Lazzeri, *Journal of Functional Biomaterials*, 2020, **11**, 62.
16. A. S. Bogdanova, A. I. Sokolova, E. R. Pavlova, D. V. Klinov and D. V. Bagrov, *Journal of Biological Physics*, 2021, **47**, 205-214.
